# Global Incidence of Frailty and Prefrailty Among Community-Dwelling Older Adults: A Systematic Review and Meta-analysis

**DOI:** 10.1001/jamanetworkopen.2019.8398

**Published:** 2019-08-02

**Authors:** Richard Ofori-Asenso, Ken L. Chin, Mohsen Mazidi, Ella Zomer, Jenni Ilomaki, Andrew R. Zullo, Danijela Gasevic, Zanfina Ademi, Maarit J. Korhonen, Dina LoGiudice, J. Simon Bell, Danny Liew

**Affiliations:** 1 School of Public Health and Preventive Medicine, Monash University, Melbourne, Victoria, Australia; 2 Melbourne Medical School, University of Melbourne, Parkville, Victoria, Australia; 3 Division of Food and Nutrition Science, Department of Biology and Biological Engineering, Chalmers University of Technology, Gothenburg, Sweden; 4 Centre for Medicine Use and Safety, Faculty of Pharmacy and Pharmaceutical Sciences, Monash University, Melbourne, Victoria, Australia; 5 Department of Health Services, Policy, and Practice, Brown University School of Public Health, Providence, Rhode Island; 6 Center of Innovation in Long Term Services and Supports, Providence Veterans Affairs Medical Center, Providence, Rhode Island; 7 Usher Institute of Population Health Sciences and Informatics, University of Edinburgh, Edinburgh, United Kingdom; 8 Institute of Biomedicine, University of Turku, Turku, Finland; 9 Department of Aged Care, Royal Melbourne Hospital and University of Melbourne, Melbourne, Victoria, Australia; 10 Centre for Research Excellence in Frailty and Healthy Ageing, University of Adelaide, Adelaide, South Australia, Australia

## Abstract

**Importance:**

Frailty is a common geriatric syndrome of significant public health importance, yet there is limited understanding of the risk of frailty development at a population level.

**Objective:**

To estimate the global incidence of frailty and prefrailty among community-dwelling adults 60 years or older.

**Data Sources:**

MEDLINE, Embase, PsycINFO, Web of Science, CINAHL Plus, and AMED (Allied and Complementary Medicine Database) were searched from inception to January 2019 without language restrictions using combinations of the keywords *frailty*, *older adults*, and *incidence*. The reference lists of eligible studies were hand searched.

**Study Selection:**

In the systematic review, 2 authors undertook the search, article screening, and study selection. Cohort studies that reported or had sufficient data to compute incidence of frailty or prefrailty among community-dwelling adults 60 years or older at baseline were eligible.

**Data Extraction and Synthesis:**

The methodological quality of included studies was assessed using The Joanna Briggs Institute’s Critical Appraisal Checklist for Prevalence and Incidence Studies. Meta-analysis was conducted using a random-effects (DerSimonian and Laird) model.

**Main Outcomes and Measures:**

Incidence of frailty (defined as new cases of frailty among robust or prefrail individuals) and incidence of prefrailty (defined as new cases of prefrailty among robust individuals), both over a specified duration.

**Results:**

Of 15 176 retrieved references, 46 observational studies involving 120 805 nonfrail (robust or prefrail) participants from 28 countries were included in this systematic review. Among the nonfrail individuals who survived a median follow-up of 3.0 (range, 1.0-11.7) years, 13.6% (13 678 of 100 313) became frail, with the pooled incidence rate being 43.4 (95% CI, 37.3-50.4; *I*^2^ = 98.5%) cases per 1000 person-years. The incidence of frailty was significantly higher in prefrail individuals than robust individuals (pooled incidence rates, 62.7 [95% CI, 49.2-79.8; *I*^2^ = 97.8%] vs 12.0 [95% CI, 8.2-17.5; *I*^2^ = 94.9%] cases per 1000 person-years, respectively; *P* for difference < .001). Among robust individuals in 21 studies who survived a median follow-up of 2.5 (range, 1.0-10.0) years, 30.9% (9974 of 32 268) became prefrail, with the pooled incidence rate being 150.6 (95% CI, 123.3-184.1; *I*^2^ = 98.9%) cases per 1000 person-years. The frailty and prefrailty incidence rates were significantly higher in women than men (frailty: 44.8 [95% CI, 36.7-61.3; *I*^2^ = 97.9%] vs 24.3 [95% CI, 19.6-30.1; *I*^2^ = 8.94%] cases per 1000 person-years; prefrailty: 173.2 [95% CI, 87.9-341.2; *I*^2^ = 99.1%] vs 129.0 [95% CI, 73.8-225.0; *I*^2^ = 98.5%] cases per 1000 person-years). The incidence rates varied by diagnostic criteria and country income level. The frailty and prefrailty incidence rates were significantly reduced when accounting for the risk of death.

**Conclusions and Relevance:**

Results of this study suggest that community-dwelling older adults are prone to developing frailty. Increased awareness of the factors that confer high risk of frailty in this population subgroup is vital to inform the design of interventions to prevent frailty and to minimize its consequences.

## Key Points

**Question:**

What is the incidence of frailty and prefrailty among community-dwelling adults 60 years or older?

**Findings:**

In this systematic review and meta-analysis involving data from more than 120 000 older adults from 28 countries, the incidence of frailty and prefrailty was estimated as 43.4 and 150.6 new cases per 1000 person-years, respectively. The frailty and prefrailty incidence rates varied by sex, diagnostic criteria, and country income level.

**Meaning:**

Results of this study suggest that the risk of developing frailty and prefrailty is high among community-living older adults; as such, appropriate interventions are needed.

## Teaser

This systematic review and meta-analysis estimates the global incidence of frailty and prefrailty among community-dwelling adults 60 years or older.

## Introduction

The increasing average life expectancy has contributed to aging of the world’s population.^1^ By 2050, approximately 21.3% of the global population will be 60 years or older,^2^ up from 9.2% in 1990. Frailty, a clinical syndrome characterized by marked vulnerability due to decline in reserve and function across multiple physiologic systems, is common among older people.^3,4^ Frailty manifests as the inability to tolerate stressful events and has been associated with adverse outcomes, such as falls,^5^ delirium,^6^ institutionalization,^7^ incident disability,^8^ and mortality.^9^ Frailty is also an independent risk factor for poor outcomes after surgery (eg, prolonged hospitalizations, increased susceptibility to deconditioning, and faster functional decline)^10^ and is associated with higher health care use^11^ and corresponding costs.^12^ There is a growing interest among stakeholders in aged care to better understand the patterns and determinants of frailty.^13^

Frailty is difficult to diagnose, particularly within primary care settings, due to its coexistence with other age-related conditions and as a result of the lack of a universally accepted clinical definition.^14,15^ There is also debate about frailty screening, especially in relation to screening eligibility, as well as where and when it should be done.^16^

Frailty phenotype and deficit accumulation are 2 main approaches to frailty assessment.^4^ Using the phenotype approach, Fried et al^17^ defined frailty as a predominantly physical condition requiring the presence of 3 or more of the following 5 components: weight loss, exhaustion, weakness, slowness, and low physical activity. However, Rockwood et al^18^ characterized frailty as an accumulation of deficits (symptoms, signs, functional impairment, and laboratory abnormalities) and stipulated that more deficits confer greater risk. These 2 frailty conceptualizations have been extensively validated and are widely used. Beyond these conceptualizations of frailty, several other definitions are present in the literature.^19^ Many definitions consider frailty to be a dynamic process with an identifiable intermediate stage, usually referred to as prefrailty.^20^

Since 2000, frailty-related research has increased exponentially.^15^ Nonetheless, the epidemiological evidence on frailty is dominated by a focus on prevalence. Incidence remains poorly understood. Although Galluzzo et al^21^ previously performed a systematic review on frailty incidence, their analysis focused on European ADVANTAGE Joint Action countries and included 6 studies, with no meta-analysis performed. With a growing worldwide interest in healthy aging,^22^ improved understanding of the incidence of frailty may help deepen the discourse around the maintenance of functional ability in old age. Therefore, we conducted a systematic review and meta-analysis to summarize the available global epidemiological data on the incidence of frailty and prefrailty among community-dwelling adults 60 years or older.

## Methods

This systematic review and meta-analysis followed the Preferred Reporting Items for Systematic Reviews and Meta-analyses^23^ (PRISMA) and Meta-analysis of Observational Studies in Epidemiology^24^ (MOOSE) reporting guidelines. The study protocol is registered at PROSPERO (CRD42019121302).^25^

### Study Eligibility Criteria

Two of us (R.O-A. and K.L.C) independently determined study eligibility, and any disagreements were resolved via consensus involving a third reviewer (D. Liew). The inclusion criteria were cohort studies that reported or had sufficient data to compute incidence of frailty or prefrailty among community-dwelling adults 60 years or older at baseline. Frailty status was considered categorically as robust, prefrail, or frail.^26^ Frailty could have been diagnosed by any method, but studies needed to specify their definition. For the Fried phenotype, individuals are often classified as robust, prefrail, or frail if 0, 1 to 2, or 3 or more of the criteria (ie, weight loss, exhaustion, weakness, slowness, and low physical activity) are met, respectively.^17^ For the deficit accumulation approach, the definitions of robust, prefrail, and frail were as specified by study authors, as has been done previously.^27,28^ Incidence of frailty was defined as new cases of frailty among robust or prefrail individuals, and incidence of prefrailty was defined as new cases of prefrailty among robust individuals, both over a specified duration. When multiple studies used the same cohort, the study with the most complete data on the largest number of participants was selected.

Exclusion criteria included studies focusing on institutionalized or hospitalized adults, residents of nursing homes (because these populations are often predominantly frail),^29^ or populations selected on the basis of an index disease. Studies reporting the mean frailty scores but without data on incidence were excluded, as were randomized clinical trials. Studies of individuals across the life span were excluded unless data were specifically available for those 60 years or older at baseline.

### Search and Selection of Studies

In the systematic review, 2 of us (R.O-A. and K.L.C.) undertook the search, article screening, and study selection. MEDLINE, Embase, PsycINFO, Web of Science, CINAHL Plus, and AMED (Allied and Complementary Medicine Database) were searched from inception to January 2019 without language restrictions using combinations of the keywords *frailty*, *older adults*, and *incidence*. eTable 1 in the Supplement lists the search terms and strategy for MEDLINE (via Ovid), which were adapted for other databases. The reference lists of eligible studies were hand searched. Conference abstracts, editorials, and meeting reports were excluded.

### Study Quality Assessment and Data Extraction

Two of us (R.O-A. and K.L.C.) evaluated each included study for methodological quality using The Joanna Briggs Institute’s Critical Appraisal Checklist for Prevalence and Incidence Studies.^30^ This checklist consists of 9 criteria, and studies were ineligible if fewer than 5 of the criteria were achieved.

The following information was collected from individual articles: study details (authors, year of publication, country, and study name), participant characteristics (sample size and percentage of women), frailty measurement method, duration of follow-up, and incidence data. Sex-stratified or age-stratified incidence data were collected, where available. Authors were contacted for additional data or clarification, when required.

### Statistical Analysis

For each study, we recorded or calculated incidence rates of frailty or prefrailty per 1000 person-years based on the event rates and the mean duration of follow-up.^27,31,32,33^ Exact methods according to the Poisson distribution were adopted to calculate 95% CIs for incidence rates.^34^ There were 2 kinds of studies, including (1) those that used a 100% survivor cohort (ie, assessed frailty status at 2 time points, excluding persons who died in-between) and (2) those that accounted for people in the cohort who died without developing frailty. Therefore, to improve the comparability of these 2 types of studies, as well as to minimize the consequences of survivorship bias,^35^ we recalculated the incidence rate in the latter studies (ie, studies that reported transition to deaths) by restricting the sample to the surviving cohort with frailty data.^27,36^

A random-effects (DerSimonian and Laird) meta-analysis was conducted using the log-transformed incidence rates and corresponding 95% CIs. The random-effects model was selected a priori due to the anticipated heterogeneity of the included studies. Statistical evidence of between-study heterogeneity was examined using the Cochran *Q* test and the *I*^2^ statistic.^37^ *I*^2^ values of 25%, 50%, and 75% were considered to be low, moderate, and high degrees of heterogeneity, respectively.^37^ The robustness of pooled estimates were assessed via leave-1-out sensitivity analyses. A study was considered to be influential if the pooled estimate without it was not within the 95% CIs of the overall pooled estimate. Sex-specific incidence data were pooled, as were the incidence rates by assessment method. To examine the extent to which the pooled incidence rates were explained by these factors, we also performed random-effects meta-regression using the following variables: measurement method (physical phenotype vs other), country income level (lower-income and middle-income country [LMIC] vs high-income country [HIC]), study region (North America, Europe, Asia, or other), person-years of follow-up (per unit increase), whether the study enrolled only elderly people 70 years or older (no vs yes), study population (mix, female only, or male only), and publication years (2009 or earlier, 2010 to 2014, or 2015 to 2019). The HICs were defined as any country with a gross national income per capita in 2017 of US $12 056 or more.^38^ Differences between subgroups were compared via a χ^2^ test. Publication bias was assessed via visual inspection of funnel plots, and statistical assessment was evaluated using the Egger test.^39^

To provide context of the burden of frailty, data on the proportion of older adults who were nonfrail were pooled using the respective study baseline data, if reported. The meta-analysis was performed using the Freeman-Tukey double arcsine transformed proportions to stabilize the variance.^40^

All analyses were performed using statistical software (Stata, version 15.0/IC; StataCorp LP). Two-tailed *P* < .05 was considered statistically significant.

## Results

### Selection Process

**Figure 1.**
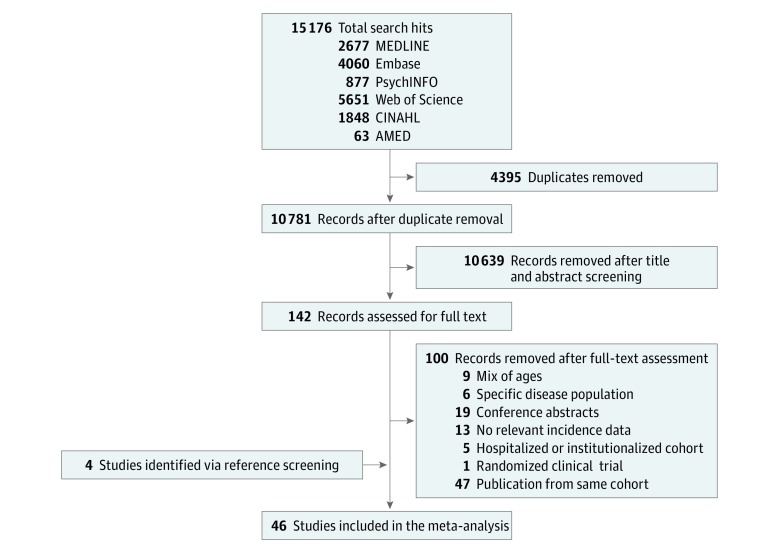
PRISMA Diagram of the Study Selection Process AMED indicates Allied and Complementary Medicine Database; PRISMA, Preferred Reporting Items for Systematic Reviews and Meta-analyses.

Of 15 176 retrieved citations, 142 articles were selected for full-text assessment (Figure 1). After full-text evaluation, 42 studies met the eligibility criteria. Four additional studies were retrieved by reference screening, resulting in a total of 46 studies (involving 48 cohorts) included in the systematic review. No study was excluded on the basis of The Joanna Briggs Institute methodological review.^30^

### Study Characteristics

The characteristics of the 46 included studies are summarized in Table 1. The studies involved 120 805 nonfrail (robust or prefrail) older adults from 28 countries. Nine studies were from Asia, 14 from North America, 2 from South America, 15 from Europe, and 4 from Australia, and 2 were cross-regional studies. eFigure 1 in the Supplement shows the geographical spread of the countries where data were collected. The median sample size across studies was 1054 (range, 44-28 181), and the median follow-up was 3.0 (range, 1.0-11.7) years. In 30 studies involving 101 259 participants, 73.3% were women. Frailty was assessed using the original or modified versions of the Fried criteria in 39 studies, 4 studies used the Frailty Index, and 1 study used both the Frailty Index and the Fried criteria, whereas 2 studies used other criteria. Among the studies using the deficit accumulation approach, the number of deficits used ranged from 20 to 44.

**Table 1.** Descriptive Characteristics of 46 Studies Included in the Systematic Review

Source (Study Region)	Study or Cohort Name	Sample Size^a^			Age Range, y	% Female	Mean Follow-up, y	Frail Diagnostic Criteria
		All	Robust	Prefrail				
Ahmad et al,^41^ 2018 (Malaysia)	NA	1677	605	1072	≥60	61.6	1.0	Fried criteria
Alencar et al,^42^ 2015 (Brazil)	NA	151	43	108	≥65	NS	1.0	Fried criteria
Ayers et al,^43^ 2017 (United States)	A: LonGenity study B: Central Control of Mobility in Aging	A: 549 B: 256	NS	NS	≥65	NS	A: 3.18 B: 1.74	Fried criteria
Baulderstone et al,^44^ 2012 (Australia)	Australian Longitudinal Study of Aging	1298	NS	NS	≥65	49.0	8.0	Fried criteria
Bentur et al,^45^ 2016 (Israel)	Members of Maccabi Healthcare Services	161	NS	NS	≥65	NS	6.0	Vulnerable Elders Survey-13
Borrat-Besson et al,^46^ 2013 (Sweden, Denmark, Germany, the Netherlands, Belgium, France, Switzerland, Austria, Spain, Italy, Poland, Czech Republic)	SHARE survey	9416	5307	4109	≥60	50.5	4.3	Fried criteria
Castrejón-Pérez et al,^47^ 2017 (Mexico)	Prospective Mexican Study of Nutritional and Psychosocial Markers of Frailty	237	NS	NS	70-95	51.5	3.0	Fried criteria
Chhetri et al,^48^ 2017 (China)	Beijing Longitudinal Study of Aging II	4378	NS	NS	≥65	NS	1.0	Frailty Index (32 deficits used: on a scale of 0-1, frailty defined as ≥0.25 deficits)
Dalrymple et al,^49^ 2013 (United States)	Cardiovascular Health Study	3459	NS	NS	≥65	100	3.0	Fried criteria
Doba et al,^50^ 2012 (Japan)	Health Research Volunteer Study	373	NS	NS	>70	54.8	5.0	Canadian Study for Health and Aging–Clinical Frailty Scale
Doi et al,^51^ 2018 (Japan)	Obu Study of Health Promotion for the Elderly	4322	1978	2344	≥65	51.9	4.0	Fried criteria
Ensrud et al,^52^ 2010 (United States)	Study of Osteoporotic Fractures	4551	NS	NS	≥65	100	4.5	Fried criteria
Espinoza et al,^53^ 2012 (United States)	San Antonio Longitudinal Study of Aging	507	209	298	≥65	NS	6.4	Fried criteria
Gale et al,^54^ 2013 (United Kingdom)	English Longitudinal Study of Ageing	2146	NS	NS	≥60	54.0	4.0	Fried criteria
García-Esquinas et al,^55^ 2015 (Spain)	Toledo Study for Healthy Aging	1289	NS	NS	≥65	58.4	3.5	Fried criteria
García-Esquinas et al,^56^ 2016 (France)	Integrated multidisciplinary approach cohort	473	NS	NS	≥65	37.8	2.0	Fried criteria
Gill et al,^57^ 2006 (United States)	Precipitating Events Project	536	167	369	≥70	NS	1.5^b^	Fried criteria
Gnjidic et al,^58^ 2012 (Australia)	Concord Health and Aging in Men Project	1242	NS	NS	≥70	0	2.0	Fried criteria
Gomes et al,^59^ 2018 (Colombia, Albania, Brazil, Canada)	International Mobility in Aging Study	1620	816	804	65-74	NS	2.0	Fried criteria
Gruenewald et al,^60^ 2009 (United States)	MacArthur Study of Successful Aging	803	440	363	70-79	55.5	3.0	Fried criteria
Hyde et al,^61^ 2016 (Australia)	Kimberley Healthy Adults Project in Indigenous Australians	44	NS	NS	≥60	NS	7.0	Frailty Index (20 deficits used: on a scale of 0-1, frailty defined as ≥0.2 deficits)
Iwasaki et al,^62^ 2018 (Japan)	Niigata Study	322	NS	NS	75	43.8	4.2	Fried criteria
Kalyani et al,^63^ 2012 (United States)	Women’s Health and Aging Study II	329	NS	NS	70-79	100	8.6	Fried criteria
Kim et al,^64^ 2017 (Japan)	Otasha-Kenshin study	684	NS	NS	≥75	100	4.0	Fried criteria
Lanziotti Azevedo da Silva et al,^65^ 2015 (Brazil)	NA	173	63	110	≥65	NS	1.1	Fried criteria
Lee et al,^66^ 2014 (Hong Kong)	Mr and Mrs OS	2893	1336	1557	≥65	48.1	2.0	Fried criteria
Liu et al,^67^ 2018 (China)	Chinese Longitudinal Healthy Longevity Survey	7601	2252	5349	65-99	NS	3.0	Frailty Index (44 deficits were used: on a scale of 0-1, robust, prefrail, and frail were defined as <0.1, 0.1-0.21, and >0.21, respectively)
Lorenzo-López et al,^68^ 2019 (Spain)	VERISAÚDE study	519	140	379	≥65	NS	1.0	Fried criteria
Ottenbacher et al,^69^ 2009 (United States)	Hispanic Established Populations Epidemiologic Studies of the Elderly	1525	737	788	≥65	42.0	10.0	Fried criteria
Pilleron et al,^70^ 2017 (France)	Three-City Bordeaux Study	1265	NS	NS	≥65	65.4	11.7	Fried criteria
Pollack et al,^71^ 2017 (United States)	Osteoporotic Fractures in Men Study	4664	2322	2342	≥65	0	4.6	Fried criteria
Potier et al,^72^ 2018 (Belgium)	NA	72	28	44	≥70	NS	1.33	Fried criteria
Ramsay et al,^73^ 2018 (United Kingdom)	British Regional Heart Study	1054	NS	NS	71-92	0	3.0	Fried criteria
Sandoval-Insausti et al,^74^ 2016 (Spain)	Seniors-ENRICA	1822	NS	NS	≥60	51.3	3.5	Fried criteria
Saum et al,^75^ 2017 (Germany)	ESTHER cohort	1446	NS	NS	≥65	NS	3.0	Fried criteria
Semba et al,^76^ 2006 (United States)	Women’s Health and Aging Study I	463	NS	NS	≥65	100	3.0	Fried criteria
Serra-Prat et al,^77^ 2017 (Spain)	NA	252	91	161	≥75	NS	1.0^b^	Fried criteria
Shah et al,^78^ 2018 (United States)	Health and Retirement Study	6073	NS	NS	≥65	56.0	4.0^c^	Fried criteria
Stephan et al,^79^ 2017 (Germany)	KORA-Age cohort study	740	218	522	≥65	NS	3.0	Frailty Index (30 items used: on a scale of 0-1 robust, prefrail, and frailty were defined as <0.08, 0.08 to <0.25, and ≥0.25, respectively)
Swiecicka et al,^80^ 2018 (Italy, Belgium, Poland, United Kingdom, Spain, Hungary, Estonia)	European Male Ageing Study	806	550	256	≥60	0	4.3	Fried criteria
Thompson et al,^81^ 2018 (Australia)	North West Adelaide Health Study	Fried criteria: 590 Frailty Index: 394	Fried criteria: 233 Frailty Index: 175	Fried criteria: 357 Frailty Index: 219	≥65	48.1	4.5	Fried criteria and Frailty Index (30 items used: on a scale of 0-1, robust, prefrail, and frailty were defined as <0.08, 0.08 to <0.25, and ≥0.25, respectively)
Tom et al,^82^ 2017 (Belgium, Canada, France, Germany, Italy, the Netherlands, Spain, United Kingdom, United States)	Global Longitudinal Study of Osteoporosis in Women	14 752	14 752	Excluded	≥60	100	2.0	Fried criteria
Trevisan et al,^83^ 2016 (Italy)	Progetto Veneto Anziani	2702	1261	1441	≥65	58.7	4.4	Fried criteria
Wang et al,^84^ 2019 (Taiwan)	NA	541	NS	NS	65-99	NS	1.0	Fried criteria
Woods et al,^85^ 2005 (United States)	Women’s Health Initiative Observational Study	28 181	NS	NS	65-79	100	3.0	Fried criteria
Zaslavsky et al,^86^ 2016 (United States)	Adult Changes in Thought Study	1848	NS	NS	≥65	57.9	4.8	Fried criteria

Abbreviations: NA, not applicable; NS, not specified.

^a^ Where available, sample size includes those who died but excludes people lost to follow-up. The total number of nonfrail people across all studies was 120 805.

^b^ Data were extracted from the follow-up duration with the most comprehensive data.

^c^ We selected the periods with the most comprehensive data as derived from a survival analysis.

In 31 studies, data on baseline proportion of older adults without frailty were available. In these studies, involving 118 411 individuals at baseline, the pooled proportion without frailty was 82.8% (95% CI, 75.8%-88.8%; *I*^2^ = 99.8%). The pooled proportion that was nonfrail was 86.5% (95% CI, 78.9%-92.7%; *I*^2^ = 99.8%) across studies that used the Fried criteria and 58.9% (95% CI, 44.2%-72.8%; *I*^2^ = 99.6%) across studies that used other criteria (*P* for difference < .001).

### Incidence of Frailty

**Figure 2.**
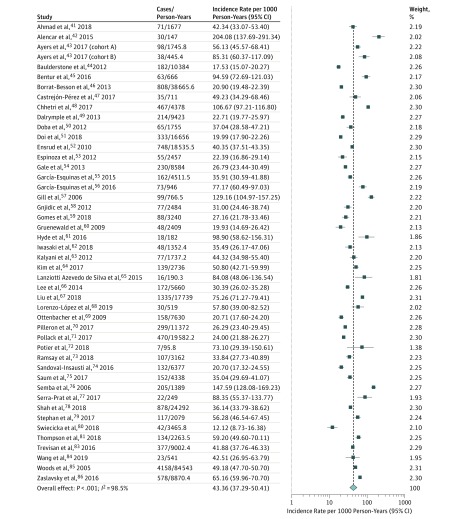
Forest Plot of the Incidence Rates (per 1000 Person-Years) of Frailty Among Community-Dwelling Older Adults Weights are from random-effects analysis. Forty-five studies were included.

To estimate the global incidence of frailty, data were included from 46 studies.^41,42,43,44,45,46,47,48,49,50,51,52,53,54,55,56,57,58,59,60,61,62,63,64,65,66,67,68,69,70,71,72,73,74,75,76,77,78,79,80,81,83,84,85,86^ Among people without frailty at baseline who survived a median follow-up of 3.0 (range, 1.0-11.7) years, 13.6% (13 678 of 100 313) became frail. The pooled incidence rate of frailty was 43.4 (95% CI, 37.3-50.4; *I*^2^ = 98.5%) cases per 1000 person-years (Figure 2). There was no evidence of publication bias as determined by funnel plot visualization (eFigure 2 in the Supplement) or via the Egger test (*P* = .48). A leave-1-out sensitivity analysis did not show a dominance of any single study (eTable 2 in the Supplement).

The pooled frailty incidence rate was 40.0 (95% CI, 34.5-48.5; *I*^2^ = 98.2%) cases per 1000 person-years when using the Fried phenotype. The pooled frailty incidence rate was 71.3 (95% CI, 56.9-89.3; *I*^2^ = 94.0%) cases per 1000 person-years when using other criteria (*P* for difference = .003).

Among 20 studies that reported transitions to death, the proportion of nonfrail people who died over a median follow-up of 4.5 years was 12.9% (5989 of 46 358). When factoring in the risk of death, the pooled incidence rate of frailty was 35.9 (95% CI, 28.0-46.1; *I*^2^ = 98.7%) cases per 1000 person-years (eFigure 3 in the Supplement). Restricting the analyses to those who survived in these 19 studies resulted in a pooled frailty incidence rate of 44.1 (95% CI, 34.0-57.2; *I*^2^ = 98.8%) cases per 1000 person-years (eFigure 4 in the Supplement).

Twenty studies reported the incidence of frailty among 19 613 people who were prefrail and 17 523 people who were robust at baseline and who survived over a median follow-up of 3.0 years. During the follow-up, 4.6% (807 of 17 523) of individuals who were robust and 18.5% (3628 of 19 613) of individuals who were prefrail developed frailty. The pooled frailty incidence rates among the robust and prefrail individuals were 12.0 (95% CI, 8.2-17.5; *I*^2^ = 94.9%) and 62.7 (95% CI, 49.2-79.8; *I*^2^ = 97.8%) cases per 1000 person-years, respectively, with the difference being statistically significant (*P* value for difference < .001).

Ten studies directly compared frailty incidence between 11 959 men and 13 870 women who survived a median follow-up of 4.0 years. Among the men and women, 9.2% (1099 of 11 959) and 15.6% (2164 of 13 870), respectively, developed frailty. The pooled incidence rates of frailty in men and women in these studies were 24.3 (95% CI, 19.6-30.1; *I*^2^ = 89.4%) and 44.8 (95% CI, 36.7-61.3; *I*^2^ = 97.9%) cases per 1000 person-years, respectively, with the difference being statistically significant (*P* value for difference = .01).

Only 2 studies^48,75^ reported age-stratified frailty incidence rate, with inconsistent age groups being used. Therefore, data were not pooled, although both studies reported consistent increases in frailty incidence with increasing age.

### Incidence of Prefrailty

**Figure 3.**
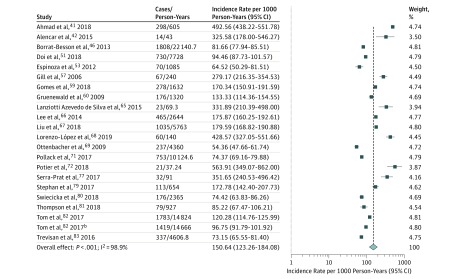
Forest Plot of the Incidence Rates (per 1000 Person-Years) of Prefrailty Among Community-Dwelling Older Adults Weights are from random-effects analysis. Twenty-one studies were included.

Twenty-one studies^41,42,46,51,53,57,59,60,65,66,67,68,69,71,72,77,79,80,81,82,83^ reported data on the global incidence of prefrailty among 32 268 community-dwelling older adults who were robust at baseline and survived a median follow-up of 2.5 (range, 1.0-10.0) years. During the follow-up, 30.9% (9974 of 32 268) became prefrail. The pooled incidence rate of prefrailty was 150.6 (95% CI, 123.3-184.1; *I*^2^ = 98.9%) cases per 1000 person-years (Figure 3). There was no evidence of publication bias as determined by visual inspection of funnel plots (eFigure 5 in the Supplement) or by means of the Egger test. A leave-1-out sensitivity analysis did not alter the results (eTable 3 in the Supplement).

The pooled incidence rate of prefrailty was 150.9 (95% CI, 120.2-182.6; *I^2^* = 98.8%) cases per 1000 person-years when using the Fried phenotype. The pooled incidence rate of prefrailty was 140.4 (95% CI, 97.2-202.9; *I^2^* = 93.4%) cases per 1000 person-years when using other criteria (*P* for difference = .52).

Among 13 studies that reported transitions to death, the proportion of robust people who died over a median follow-up of 4.0 years was 7.8% (1253 of 16 134). When factoring in the risk of death, the pooled incidence rate of prefrailty was 110.6 (95% CI, 84.8-144.2; *I*^2^ = 98.9%) cases per 1000 person-years (eFigure 6 in the Supplement). Restricting the analyses to those who survived in these 13 studies resulted in a pooled prefrailty incidence rate of 122.7 (95% CI, 95.7-157.5; *I*^2^ = 98.7%) cases per 1000 person-years (eFigure 7 in the Supplement).

Four studies directly compared incidence of prefrailty among 4003 men and 3655 women who survived a median follow-up of 4.2 years. In all, 32.6% (1305 of 4003) of the men and 40.1% (1465 of 3655) of the women became prefrail, at a pooled incidence rate of 129.0 (95% CI, 73.8-225.0; *I*^2^ = 98.5%) and 173.2 (95% CI, 87.9-341.2; *I*^2^ = 99.1%) cases per 1000 person-years, respectively (*P* for difference = .12). No study reported age-stratified prefrailty incidence data.

### Meta-regression

In the multivariable random-effects meta-regression, measuring frailty as a physical phenotype was associated with higher incidence than using other methods (adjusted odds ratio [aOR], 1.48; 95% CI, 1.02-2.15), although no statistically significant difference was observed for prefrailty incidence (Table 2). Study region was not significantly associated with frailty and prefrailty incidence, but HICs were associated with a lower incidence of frailty (aOR, 0.63; 95% CI, 0.42-0.95) and prefrailty (aOR, 0.30; 95% CI, 0.21-0.84) compared with LMICs. Studies published after 2009 were associated with lower frailty incidence. The variables included in the multivariable models collectively explained about 63.9% and 38.1% of the between-study variance for frailty and prefrailty incidence, respectively.

**Table 2.** Results of Univariable and Multivariable Random-Effects Meta-regression of the Sources of Between-Study Heterogeneity

Variable	Univariable			Multivariable		
	OR (95% CI)	*P* Value	Adjusted *R*^2^, %	aOR (95% CI)	*P* Value	Adjusted *R*^2^, %
**Incidence of Frailty**						
Measurement method						63.9
Physical phenotype	1 [Reference]	NA	10.1	1 [Reference]	NA	
Other	1.78 (1.09-2.89)	.02		1.48 (1.02-2.15)	.03	
Country income level						
LMIC	1 [Reference]	NA	7.6	1 [Reference]	NA	
HIC	0.59 (0.36-0.97)	.04		0.63 (0.42-0.95)	.03	
Study region						
North America	1 [Reference]	NA	1.2	1 [Reference]	NA	
Europe	0.83 (0.52-1.32)	.43		0.88 (0.63-1.24)	.45	
Asia	0.99 (0.59-1.67)	.98		0.74 (0.50-1.10)	.13	
Other	1.45 (0.84-2.50)	.18		1.23 (0.82-1.84)	.31	
Person-years of follow-up per unit increase	0.99 (0.99-1.00)	.17	1.8	0.99 (0.99-0.99)	.02	
Enrolled only elderly people (≥70 y)						
No	1 [Reference]	NA	−2.1	1 [Reference]	NA	
Yes	1.08 (0.69-1.67)	.34		1.18 (0.85-1.63)	.31	
Study population						
Mix	1 [Reference]	NA	5.8	1 [Reference]	NA	
Female only	1.13 (0.64-2.00)	.67		1.14 (0.72-1.79)	.57	
Male only	0.52 (0.27-0.97)	.04		0.55 (0.35-0.87)	.01	
Publication years						
2009 Or earlier	1 [Reference]	<.001	29.1	1 [Reference]	NA	
2010-2014	0.27 (0.14-0.54)	<.001		0.24 (0.14-0.44)	<.001	
2015-2019	0.50 (0.27-0.95)	.03		0.42 (0.22-0.77)	.007	
**Incidence of Prefrailty**						
Measurement method						38.1
Physical phenotype	1 [Reference]	NA	−1.7	1 [Reference]	NA	
Other	0.65 (0.23-1.79)	.40		0.45 (0.18-1.16)	NA	
Country income level						
LMIC	1 [Reference]	NA	18.4	1 [Reference]	NA	
HIC	0.39 (0.17-0.90)	.03		0.30 (0.21-0.84)	.03	
Study region						
North America	1 [Reference]	NA	−10.8	1 [Reference]	NA	
Europe	1.61 (0.63-4.10)	.24		1.66 (0.62-4.49)	.28	
Asia	1.91 (0.63-5.82)	.24		1.14 (0.33-3.90)	.82	
Other	1.22 (0.39-3.79)	.72		0.56 (0.15-2.15)	.36	
Person-years of follow-up per unit increase	1.00 (0.99-1.00)	.07	11.2	1.00 (0.99-1.00)	.21	
Enrolled only elderly people (≥70 y)						
No	1 [Reference]	NA	1.8	1 [Reference]	NA	
Yes	1.76 (0.64-4.81)	.26		1.40 (0.44-4.47)	.54	
Study population						
Mix	1 [Reference]	NA	−0.1	1 [Reference]	NA	
Female only	1.47 (0.44-4.93)	.51		1.02 (0.21-4.89)	.98	
Male only	0.69 (0.14-3.50)	.64		0.49 (0.13-1.81)	.25	
Publication years						
2009 Or earlier	1 [Reference]	NA	2.8	1 [Reference]	NA	
2010-2014	0.33 (0.06-1.95)	.21		0.49 (0.09-2.86)	.39	
2015-2019	0.56 (0.11-2.99)	.48		0.76 (0.11-5.25)	.76	

Abbreviations: aOR, adjusted odds ratio; HIC, high-income country; LMIC, lower-income and middle-income country; NA, not applicable; OR, odds ratio.

## Discussion

We performed a systematic review and meta-analysis to estimate the incidence of frailty and prefrailty among community-dwelling older adults. Our results indicate the following: (1) frailty and prefrailty incidence rates were approximately 43 and 151 new cases per 1000 person-years, respectively; (2) the incidence of frailty and prefrailty was higher in women than men; and (3) the incidence of frailty and prefrailty varied by frailty measurement method used and by country income level.

Although not necessarily synonymous with aging, frailty is highly prevalent among older people.^3,4^ Our pooled baseline data suggested that approximately 1 in 6 community-dwelling older people may have frailty. Frailty has been associated with adverse health outcomes, such as falls, disability, and death, as well as increased use of health care resources.^8,9,12^ Therefore, efforts to reduce the burden of frailty could have substantial public health consequences.

Prevention of frailty requires a sound understanding of the risk factors. For example, it has been demonstrated that individual chronic diseases (eg, cancers, type 2 diabetes,^63,66,71^ and depression,^77,85,87^ or their co-occurrence [ie, multimorbidity]) have been shown to increase the risk of frailty.^88,89^ With an estimated 66% of older people having at least 2 chronic medical conditions,^90^ effective preventive strategies are paramount to reduce overall disease burden. The rising prevalence of obesity among older adults^91,92^ needs greater attention because this condition, particularly abdominal obesity, may increase the risk of frailty through the association with proinflammatory processes, insulin resistance, fat infiltration of skeletal muscles, and hormonal alterations.^93,94^ Many other sociodemographic, physical, biological, lifestyle (eg, smoking), and psychological factors may equally contribute to the development of frailty and thus require tailored solutions in different settings.^95,96,97,98^

We found a higher incidence of frailty and prefrailty in LMICs than HICs in our study, which is consistent with prior observations of significantly higher prevalence of frailty and prefrailty in LMICs compared with HICs.^99^ Some studies^59,87,100^ found that high income and educational levels and greater access to and quality of health care confer lower frailty risk, which may partly explain the disparity in frailty incidence between LMICs and HICs, presenting opportunity to prevent or delay the onset of chronic pathologies associated with increased risk of frailty.^88,101^

Our meta-analysis suggests higher incidence of frailty and prefrailty in women than men. Previous studies have shown consistently higher prevalence rates^3,99^ and frailty scores^102^ among women than men across all age groups. The sex differences may be attributable to both biological and socioeconomic factors. Nonetheless, women have been found to better tolerate frailty, as evidenced by lower mortality rates at any frailty level or age, suggesting the existence of a male-female health-survival paradox.^102^

To date, several interventions incorporating exercise, nutrition, cognitive training, geriatric assessment, hormone therapy, and management and prehabilitation have been evaluated for their effectiveness at delaying or reversing frailty.^103,104,105,106,107^ Most of these interventions have demonstrated feasibility, with adherence rates of about 70%.^103^ However, a recent systematic review reported that, among the available primary care interventions to delay or reverse frailty, strength training and protein supplementation ranked highest in terms of relative effectiveness and ease of implementation.^108^ Conversely, mild-intensity mixed exercises, as well as educational or health promotion activities, typically were in the midzone for both relative effectiveness and ease of implementation, whereas comprehensive geriatric assessments and home visits were ranked mid to low for both relative effectiveness and ease of implementation. In general, interventions targeting behavioral change ranked low in relative effectiveness and at the midzone for ease of implementation.^108^ However, it needs emphasizing that most interventions have been tested in people who were frail or prefrail.^103,108^ Our meta-analysis showed that, among people who were robust, there were approximately 12 and 151 new cases of frailty and prefrailty per 1000 person-years, respectively, suggesting that interventions aimed at preventing frailty and prefrailty in robust populations could be important.

The lower pooled incidence when frailty was defined as a physical phenotype compared with when a broad phenotype was used is consistent with prior meta-analyses that have demonstrated higher frailty prevalence when using broad definitions vs the physical phenotype.^3,99^ Other studies^3^ have shown considerable variability in the literature regarding the use of the deficit accumulation approach (as also observed in the present study), thus contributing to wide estimates of frailty burden. Therefore, a harmonized definition of frailty may be useful.

### Limitations and Future Directions

Our study had some limitations. There was substantial heterogeneity of the included studies. Nonetheless, heterogeneity is often inevitable in meta-analyses of observational studies, and it does not necessarily invalidate the findings.^109^ We decided a priori to pool incidence data across studies that met our inclusion criteria. Furthermore, potential sources of heterogeneity were investigated via subgroup and random-effects meta-regression, which showed considerable heterogeneity in incidence rates by frailty measurement method, country income level, and publication years of studies. Meta-analysis of incidence data is also complicated by variable duration of follow-up. We sought to overcome this by estimating person-years on the basis of the median follow-up duration. While this method is considered robust and is widely applied in the literature,^27,31,32,33^ a more precise approach would have required the use of the actual data on person-years, which were unavailable in more than 90% of studies. While frailty incidence varies by age, we could not perform age-stratified analysis due to limited data, and we were unable to account for the influence of the mean age of participants in the individual studies in the regression models due to poor reporting. People who develop frailty or prefrailty may regress^27,36^; however, the present analysis does not incorporate regression rates. Finally, our abstract screening may have missed relevant studies in which frailty was not the main focus, but which contained information on the incidence of frailty (eg, frailty as a covariate).

Overall, the study results reiterate the need for regular screening programs to assess older people’s vulnerability to frailty development so that appropriate interventions can be implemented in a timely manner.^16^ For example, frailty assessment could be considered as part of routine health screening or could be instituted as a part of the core services delivered to older people within primary health care and general practice settings.^41^ Because not all older people develop frailty, future studies should examine protective factors against frailty so as to inform preventive strategies. Our data could also inform health care planning and design of preventive strategies. However, the inequality in the availability of frailty data according to geographical locations requires attention because it hampers the opportunity to reliably forecast the future trajectory of the global burden of frailty, which is needed to inform efficient planning and resource allocation, mindful of the growing aging population.^21^

## Conclusions

There is a high risk of frailty among community-dwelling older adults, and we observed that the incidence of frailty varies by sex, region, country income level, and diagnostic criteria used. It is imperative to improve understanding of the factors that confer increased risk of frailty. This will help inform the design of interventions to prevent frailty or minimize its negative consequences on health.

## Notes

Supplement. **eTable 1.** Search Sequence for Ovid Medline Which Was Subsequently Adapted for Other Databases **eFigure 1.** A Map Showing the Geographical Spread of the Countries From Which Data Were Collected **eFigure 2.** Funnel Plot of Incidence Rates of Frailty Among Community-Dwelling Older Adults **eFigure 3.** Forest Plot of Incidence Rates of Frailty Among Community-Dwelling Older Adults When Factoring in Progression to Deaths in Studies With Death Data **eFigure 4.** Forest Plot of Incidence Rates of Frailty Among Community-Dwelling Older Adults When Limiting to Survivors in Studies With Death Data **eFigure 5.** Funnel Plot of Incidence Rates of Pre-frailty Among Community-Dwelling Older Adults **eFigure 6.** Forest Plot of Incidence Rates of Pre-frailty Among Community-Dwelling Older Adults When Factoring in Progression to Deaths in Studies With Death Data **eFigure 7.** Forest Plot of Incidence Rates of Pre-frailty Among Community-Dwelling Older Adults When Limiting to Survivors in Studies With Death Data **eTable 2.** Estimates From Leave-One-Out Sensitivity Analyses for the Incidence of Frailty **eTable 3.** Estimates of Leave-One-Out Sensitivity Analyses for the Incidence of Pre-frailty **eReferences.**

## References

[1] Sander M, Oxlund B, Jespersen A, The challenges of human population ageing. Age Ageing. 2015;44(2):-. doi:10.1093/ageing/afu189 25452294 PMC4339729

[2] United Nations Department of Economic and Social Affairs (DESA)/Population Division World Population Prospects 2019. https://population.un.org/wpp/Download/Standard/Population/. Accessed January 26, 2019.

[3] Collard RM, Boter H, Schoevers RA, Oude Voshaar RC Prevalence of frailty in community-dwelling older persons: a systematic review. J Am Geriatr Soc. 2012;60(8):1487-1492. doi:10.1111/j.1532-5415.2012.04054.x 22881367

[4] Clegg A, Young J, Iliffe S, Rikkert MO, Rockwood K Frailty in elderly people. Lancet. 2013;381(9868):752-762. doi:10.1016/S0140-6736(12)62167-9 23395245 PMC4098658

[5] Cheng MH, Chang SF Frailty as a risk factor for falls among community dwelling people: evidence from a meta-analysis. J Nurs Scholarsh. 2017;49(5):529-536. doi:10.1111/jnu.12322 28755453

[6] Persico I, Cesari M, Morandi A, Frailty and delirium in older adults: a systematic review and meta-analysis of the literature. J Am Geriatr Soc. 2018;66(10):2022-2030. doi:10.1111/jgs.15503 30238970

[7] Kojima G Frailty as a predictor of nursing home placement among community-dwelling older adults: a systematic review and meta-analysis. J Geriatr Phys Ther. 2018;41(1):42-48. doi:10.1519/JPT.0000000000000097 27341327

[8] Kojima G Frailty as a predictor of disabilities among community-dwelling older people: a systematic review and meta-analysis. Disabil Rehabil. 2017;39(19):1897-1908. doi:10.1080/09638288.2016.1212282 27558741

[9] Kojima G, Iliffe S, Walters K Frailty Index as a predictor of mortality: a systematic review and meta-analysis. Age Ageing. 2018;47(2):193-200. doi:10.1093/ageing/afx162 29040347

[10] Lin HS, Watts JN, Peel NM, Hubbard RE Frailty and post-operative outcomes in older surgical patients: a systematic review. BMC Geriatr. 2016;16(1):157. doi:10.1186/s12877-016-0329-8 27580947 PMC5007853

[11] Kojima G Frailty as a predictor of emergency department utilization among community-dwelling older people: a systematic review and meta-analysis. J Am Med Dir Assoc. 2019;20(1):103-105. doi:10.1016/j.jamda.2018.10.004 30470576

[12] Bock JO, König HH, Brenner H, Associations of frailty with health care costs: results of the ESTHER cohort study. BMC Health Serv Res. 2016;16:128. doi:10.1186/s12913-016-1360-3 27074800 PMC4831082

[13] Gwyther H, Shaw R, Jaime Dauden EA, Understanding frailty: a qualitative study of European healthcare policy-makers’ approaches to frailty screening and management. BMJ Open. 2018;8(1):e018653. doi:10.1136/bmjopen-2017-018653 29331967 PMC5781160

[14] MorleyJE Frailty: diagnosis and management. J Nutr Health Aging. 2011;15(8):667-670. doi:10.1007/s12603-011-0338-4 21968862

[15] Buckinx F, Rolland Y, Reginster JY, Ricour C, Petermans J, Bruyère O Burden of frailty in the elderly population: perspectives for a public health challenge. Arch Public Health. 2015;73(1):19. doi:10.1186/s13690-015-0068-x 25866625 PMC4392630

[16] Ambagtsheer RC, Beilby JJ, Visvanathan R, Dent E, Yu S, Braunack-Mayer AJ Should we screen for frailty in primary care settings? a fresh perspective on the frailty evidence base: a narrative review. Prev Med. 2019;119:63-69. doi:10.1016/j.ypmed.2018.12.020 30594533

[17] Fried LP, Tangen CM, Walston J, ; Cardiovascular Health Study Collaborative Research Group Frailty in older adults: evidence for a phenotype. J Gerontol A Biol Sci Med Sci. 2001;56(3):M146-M156. doi:10.1093/gerona/56.3.M146 11253156

[18] Rockwood K, Stadnyk K, MacKnight C, McDowell I, Hébert R, Hogan DB A brief clinical instrument to classify frailty in elderly people. Lancet. 1999;353(9148):205-206. doi:10.1016/S0140-6736(98)04402-X 9923878

[19] Dent E, Kowal P, Hoogendijk EO Frailty measurement in research and clinical practice: a review. Eur J Intern Med. 2016;31:3-10. doi:10.1016/j.ejim.2016.03.007 27039014

[20] Lang PO, Michel JP, Zekry D Frailty syndrome: a transitional state in a dynamic process. Gerontology. 2009;55(5):539-549. doi:10.1159/000211949 19346741

[21] Galluzzo L, O’Caoimh R, Rodríguez-Laso Á, ; Work Package 5 of the Joint Action ADVANTAGE Incidence of frailty: a systematic review of scientific literature from a public health perspective. Ann Ist Super Sanita. 2018;54(3):239-245. 30284551 10.4415/ANN_18_03_11

[22] Kaeberlein M, Rabinovitch PS, Martin GM Healthy aging: the ultimate preventative medicine. Science. 2015;350(6265):1191-1193. doi:10.1126/science.aad3267 26785476 PMC4793924

[23] Moher D, Liberati A, Tetzlaff J, Altman DG; PRISMA Group Preferred Reporting Items for Systematic Reviews and Meta-analyses: the PRISMA statement. BMJ. 2009;339:b2535. doi:10.1136/bmj.b2535 19622551 PMC2714657

[24] Stroup DF, Berlin JA, Morton SC, ; Meta-analysis of Observational Studies in Epidemiology (MOOSE) Group. Meta-analysis of Observational Studies in Epidemiology: a proposal for reporting. JAMA. 2000;283(15):2008-2012. doi:10.1001/jama.283.15.2008 10789670

[25] PROSPERO Incidence of Frailty Among Community-Dwelling Older Adults. CRD42019121302. https://www.crd.york.ac.uk/prospero/display_record.php?RecordID=121302. Accessed January 26, 2019.

[26] Xue QL The frailty syndrome: definition and natural history. Clin Geriatr Med. 2011;27(1):1-15. doi:10.1016/j.cger.2010.08.009 21093718 PMC3028599

[27] Ofori-Asenso R, Lee Chin K, Mazidi M, Natural regression of frailty among community-dwelling older adults: a systematic review and meta-analysis. Gerontologist. 2019;gnz064. doi:10.1093/geront/gnz064 31115434

[28] Aguayo GA, Donneau AF, Vaillant MT, Agreement between 35 published frailty scores in the general population. Am J Epidemiol. 2017;186(4):420-434. doi:10.1093/aje/kwx061 28633404 PMC5860330

[29] Kojima G Prevalence of frailty in nursing homes: a systematic review and meta-analysis. J Am Med Dir Assoc. 2015;16(11):940-945. doi:10.1016/j.jamda.2015.06.025 26255709

[30] Munn Z, Moola S, Lisy K, Riitano D, Tufanaru C Methodological guidance for systematic reviews of observational epidemiological studies reporting prevalence and cumulative incidence data. Int J Evid Based Healthc. 2015;13(3):147-153. doi:10.1097/XEB.0000000000000054 26317388

[31] Sikkema M, de Jonge PJ, Steyerberg EW, Kuipers EJ Risk of esophageal adenocarcinoma and mortality in patients with Barrett’s esophagus: a systematic review and meta-analysis. Clin Gastroenterol Hepatol. 2010;8(3):235-244. doi:10.1016/j.cgh.2009.10.010 19850156

[32] Tansel A, Katz LH, El-Serag HB, Incidence and determinants of hepatocellular carcinoma in autoimmune hepatitis: a systematic review and meta-analysis. Clin Gastroenterol Hepatol. 2017;15(8):1207-1217.e4. doi:10.1016/j.cgh.2017.02.006 28215616 PMC5522646

[33] Yousef F, Cardwell C, Cantwell MM, Galway K, Johnston BT, Murray L The incidence of esophageal cancer and high-grade dysplasia in Barrett’s esophagus: a systematic review and meta-analysis. Am J Epidemiol. 2008;168(3):237-249. doi:10.1093/aje/kwn121 18550563

[34] Sutton A, Abrams K, Jones D, Sheldon T, Song F Methods for Meta-analysis in Medical Research. London, United Kingdom: Wiley; 2000.

[35] Delgado-Rodríguez M, Llorca J Bias. J Epidemiol Community Health. 2004;58(8):635-641. doi:10.1136/jech.2003.008466 15252064 PMC1732856

[36] Kojima G, Taniguchi Y, Iliffe S, Jivraj S, Walters K Transitions between frailty states among community-dwelling older people: a systematic review and meta-analysis. Ageing Res Rev. 2019;50:81-88. doi:10.1016/j.arr.2019.01.010 30659942

[37] Higgins JP, Thompson SG, Deeks JJ, Altman DG Measuring inconsistency in meta-analyses. BMJ. 2003;327(7414):557-560. doi:10.1136/bmj.327.7414.557 12958120 PMC192859

[38] World Bank World Bank country and lending groups. https://datahelpdesk.worldbank.org/knowledgebase/articles/906519-world-bank-country-and-lending-groups. Published 2019. Accessed January 29, 2019.

[39] Egger M, Davey Smith G, Schneider M, Minder C Bias in meta-analysis detected by a simple, graphical test. BMJ. 1997;315(7109):629-634. doi:10.1136/bmj.315.7109.629 9310563 PMC2127453

[40] Barendregt JJ, Doi SA, Lee YY, Norman RE, Vos T Meta-analysis of prevalence. J Epidemiol Community Health. 2013;67(11):974-978. doi:10.1136/jech-2013-203104 23963506

[41] Ahmad NS, Hairi NN, Said MA, Prevalence, transitions and factors predicting transition between frailty states among rural community-dwelling older adults in Malaysia. PLoS One. 2018;13(11):e0206445. doi:10.1371/journal.pone.0206445 30395649 PMC6218037

[42] Alencar MA, Dias JMD, Figueiredo LC, Dias RC Transitions in frailty status in community-dwelling older adults. Top Geriatr Rehabil. 2015;31(2):105-112. doi:10.1097/TGR.0000000000000055

[43] Ayers E, Shapiro M, Holtzer R, Barzilai N, Milman S, Verghese J Symptoms of apathy independently predict incident frailty and disability in community-dwelling older adults. J Clin Psychiatry. 2017;78(5):e529-e536. doi:10.4088/JCP.15m10113 28406265 PMC5592638

[44] Baulderstone L, Yaxley A, Luszcz M, Miller M Diet liberalisation in older Australians decreases frailty without increasing the risk of developing chronic disease. J Frailty Aging. 2012;1(4):174-182. 27093318 10.14283/jfa.2012.27

[45] Bentur N, Sternberg SA, Shuldiner J Frailty transitions in community dwelling older people. Isr Med Assoc J. 2016;18(8):449-453. 28471574

[46] Borrat-Besson C, Ryser V, Wernli B Transitions between frailty states: a European comparison In: Börsch-Supan A, Brandt M, Litwin H, Weber G, eds. Active Ageing and Solidarity Between Generations in Europe: First Results From SHARE After the Economic Crisis. Gottingen, Germany: Hubert & Co; 2013:175-185. doi:10.1515/9783110295467.175

[47] Castrejón-Pérez RC, Jiménez-Corona A, Bernabé E, Oral disease and 3-year incidence of frailty in Mexican older adults. J Gerontol A Biol Sci Med Sci. 2017;72(7):951-957. 28329793 10.1093/gerona/glw201

[48] Chhetri JK, Zheng Z, Xu X, Ma C, Chan P The prevalence and incidence of frailty in pre-diabetic and diabetic community-dwelling older population: results from Beijing Longitudinal Study of Aging II (BLSA-II). BMC Geriatr. 2017;17(1):47. doi:10.1186/s12877-017-0439-y 28178934 PMC5299771

[49] Dalrymple LS, Katz R, Rifkin DE, Kidney function and prevalent and incident frailty. Clin J Am Soc Nephrol. 2013;8(12):2091-2099. doi:10.2215/CJN.02870313 24178972 PMC3848393

[50] Doba N, Tokuda Y, Goldstein NE, Kushiro T, Hinohara S A pilot trial to predict frailty syndrome: the Japanese Health Research Volunteer Study. Exp Gerontol. 2012;47(8):638-643. doi:10.1016/j.exger.2012.05.016 22664579

[51] Doi T, Makizako H, Tsutsumimoto K, Transitional status and modifiable risk of frailty in Japanese older adults: a prospective cohort study. Geriatr Gerontol Int. 2018;18(11):1562-1566. doi:10.1111/ggi.13525 30225955

[52] Ensrud KE, Ewing SK, Fredman L, ; Study of Osteoporotic Fractures Research Group Circulating 25-hydroxyvitamin D levels and frailty status in older women. J Clin Endocrinol Metab. 2010;95(12):5266-5273. doi:10.1210/jc.2010-2317 21131545 PMC2999979

[53] Espinoza SE, Jung I, Hazuda H Frailty transitions in the San Antonio Longitudinal Study of Aging. J Am Geriatr Soc. 2012;60(4):652-660. doi:10.1111/j.1532-5415.2011.03882.x 22316162 PMC3325321

[54] Gale CR, Baylis D, Cooper C, Sayer AA Inflammatory markers and incident frailty in men and women: the English Longitudinal Study of Ageing. Age (Dordr). 2013;35(6):2493-2501. doi:10.1007/s11357-013-9528-9 23543263 PMC3751755

[55] García-Esquinas E, José García-García F, León-Muñoz LM, Obesity, fat distribution, and risk of frailty in two population-based cohorts of older adults in Spain. Obesity (Silver Spring). 2015;23(4):847-855. doi:10.1002/oby.21013 25683024

[56] García-Esquinas E, Rahi B, Peres K, Consumption of fruit and vegetables and risk of frailty: a dose-response analysis of 3 prospective cohorts of community-dwelling older adults. Am J Clin Nutr. 2016;104(1):132-142. doi:10.3945/ajcn.115.125781 27194305

[57] Gill TM, Gahbauer EA, Allore HG, Han L Transitions between frailty states among community-living older persons. Arch Intern Med. 2006;166(4):418-423. doi:10.1001/archinte.166.4.418 16505261

[58] Gnjidic D, Hilmer SN, Blyth FM, High-risk prescribing and incidence of frailty among older community-dwelling men. Clin Pharmacol Ther. 2012;91(3):521-528. doi:10.1038/clpt.2011.258 22297385

[59] Gomes CDS, Guerra RO, Wu YY, Social and economic predictors of worse frailty status occurrence across selected countries in North and South America and Europe. Innov Aging. 2018;2(3):igy037. doi:10.1093/geroni/igy037 30569024 PMC6295000

[60] Gruenewald TL, Seeman TE, Karlamangla AS, Sarkisian CA Allostatic load and frailty in older adults. J Am Geriatr Soc. 2009;57(9):1525-1531. doi:10.1111/j.1532-5415.2009.02389.x 19682116 PMC3650612

[61] Hyde Z, Flicker L, Smith K, Prevalence and incidence of frailty in Aboriginal Australians, and associations with mortality and disability. Maturitas. 2016;87:89-94. doi:10.1016/j.maturitas.2016.02.013 27013294

[62] Iwasaki M, Yoshihara A, Sato M, Dentition status and frailty in community-dwelling older adults: a 5-year prospective cohort study. Geriatr Gerontol Int. 2018;18(2):256-262. doi:10.1111/ggi.13170 28944598

[63] Kalyani RR, Tian J, Xue QL, Hyperglycemia and incidence of frailty and lower extremity mobility limitations in older women. J Am Geriatr Soc. 2012;60(9):1701-1707. doi:10.1111/j.1532-5415.2012.04099.x 22882211 PMC4144067

[64] Kim M, Suzuki T, Kojima N, Association between serum β_2_-microglobulin levels and prevalent and incident physical frailty in community-dwelling older women. J Am Geriatr Soc. 2017;65(4):e83-e88. doi:10.1111/jgs.14733 28140452

[65] Lanziotti Azevedo da Silva S, Campos Cavalcanti Maciel Á, de Sousa Máximo Pereira L, Domingues Dias JM, Guimarães de Assis M, Corrêa Dias R Transition patterns of frailty syndrome in community-dwelling elderly individuals: a longitudinal study. J Frailty Aging. 2015;4(2):50-55. 27032045 10.14283/jfa.2015.43

[66] Lee JSW, Auyeung TW, Leung J, Kwok T, Woo J Transitions in frailty states among community-living older adults and their associated factors. J Am Med Dir Assoc. 2014;15(4):281-286. doi:10.1016/j.jamda.2013.12.002 24534517

[67] Liu ZY, Wei YZ, Wei LQ, Frailty transitions and types of death in Chinese older adults: a population-based cohort study. Clin Interv Aging. 2018;13:947-956. doi:10.2147/CIA.S157089 29805253 PMC5960243

[68] Lorenzo-López L, López-López R, Maseda A, Buján A, Rodríguez-Villamil JL, Millán-Calenti JC Changes in frailty status in a community-dwelling cohort of older adults: the VERISAÚDE study. Maturitas. 2019;119:54-60. doi:10.1016/j.maturitas.2018.11.006 30502751

[69] Ottenbacher KJ, Graham JE, Al Snih S, Mexican Americans and frailty: findings from the Hispanic established populations epidemiologic studies of the elderly. Am J Public Health. 2009;99(4):673-679. doi:10.2105/AJPH.2008.143958 19197079 PMC2661466

[70] Pilleron S, Ajana S, Jutand MA, Dietary patterns and 12-year risk of frailty: results from the Three-City Bordeaux Study. J Am Med Dir Assoc. 2017;18(2):169-175. doi:10.1016/j.jamda.2016.09.014 27847264

[71] Pollack LR, Litwack-Harrison S, Cawthon PM, Patterns and predictors of frailty transitions in older men: the Osteoporotic Fractures in Men Study. J Am Geriatr Soc. 2017;65(11):2473-2479. doi:10.1111/jgs.15003 28873220 PMC5681371

[72] Potier F, Degryse JM, Bihin B, Health and frailty among older spousal caregivers: an observational cohort study in Belgium. BMC Geriatr. 2018;18(1):291. doi:10.1186/s12877-018-0980-3 30477431 PMC6258488

[73] Ramsay SE, Papachristou E, Watt RG, Influence of poor oral health on physical frailty: a population‐based cohort study of older British men. J Am Geriatr Soc. 2018;66(3):473-479. doi:10.1111/jgs.15175 29266166 PMC5887899

[74] Sandoval-Insausti H, Pérez-Tasigchana RF, López-García E, García-Esquinas E, Rodríguez-Artalejo F, Guallar-Castillón P Macronutrients intake and incident frailty in older adults: a prospective cohort study. J Gerontol A Biol Sci Med Sci. 2016;71(10):1329-1334. doi:10.1093/gerona/glw033 26946103

[75] Saum KU, Schöttker B, Meid AD, Is polypharmacy associated with frailty in older people? results from the ESTHER cohort study. J Am Geriatr Soc. 2017;65(2):e27-e32. doi:10.1111/jgs.14718 28024089

[76] Semba RD, Bartali B, Zhou J, Blaum C, Ko CW, Fried LP Low serum micronutrient concentrations predict frailty among older women living in the community. J Gerontol A Biol Sci Med Sci. 2006;61(6):594-599. doi:10.1093/gerona/61.6.594 16799142

[77] Serra-Prat M, Papiol M, Vico J, Palomera E, Arús M, Cabré M Incidence and risk factors for frailty in the community-dwelling elderly population: a two-year follow-up cohort study. J Gerontol Geriatr Res. 2017;6(6):452. doi:10.4172/2167-7182.1000452

[78] Shah M, Paulson D, Nguyen V Alcohol use and frailty risk among older adults over 12 years: the Health and Retirement Study. Clin Gerontol. 2018;41(4):315-325. doi:10.1080/07317115.2017.1364681 28990855

[79] Stephan AJ, Strobl R, Holle R, Male sex and poverty predict abrupt health decline: deficit accumulation patterns and trajectories in the KORA-Age cohort study. Prev Med. 2017;102:31-38. doi:10.1016/j.ypmed.2017.06.032 28663079

[80] Swiecicka A, Eendebak RJAH, Lunt M, ; European Male Ageing Study Group Reproductive hormone levels predict changes in frailty status in community-dwelling older men: European Male Ageing Study prospective data. J Clin Endocrinol Metab. 2018;103(2):701-709. doi:10.1210/jc.2017-01172 29186457 PMC5800832

[81] Thompson MQ, Theou O, Adams RJ, Tucker GR, Visvanathan R Frailty state transitions and associated factors in South Australian older adults. Geriatr Gerontol Int. 2018;18(11):1549-1555. doi:10.1111/ggi.13522 30221449

[82] Tom S, Wyman A, Woods N, Regional differences in incident prefrailty and frailty. J Womens Health (Larchmt). 2017;26(9):992-998. doi:10.1089/jwh.2016.6041

[83] Trevisan C, Veronese N, Maggi S, Marital status and frailty in older people: gender differences in the Progetto Veneto Anziani longitudinal study. J Womens Health (Larchmt). 2016;25(6):630-637. doi:10.1089/jwh.2015.5592 26845424

[84] Wang MC, Li TC, Li CI, Frailty, transition in frailty status and all-cause mortality in older adults of a Taichung community-based population. BMC Geriatr. 2019;19(1):26. doi:10.1186/s12877-019-1039-9 30691410 PMC6348637

[85] Woods NF, LaCroix AZ, Gray SL, ; Women’s Health Initiative Frailty: emergence and consequences in women aged 65 and older in the Women’s Health Initiative Observational Study [published correction appears in *J Am Geriatr Soc*. 2017;65(7):1631-1632]. J Am Geriatr Soc. 2005;53(8):1321-1330. 16078957 10.1111/j.1532-5415.2005.53405.x

[86] Zaslavsky O, Walker RL, Crane PK, Gray SL, Larson EB Glucose levels and risk of frailty. J Gerontol A Biol Sci Med Sci. 2016;71(9):1223-1229. doi:10.1093/gerona/glw024 26933160 PMC4978362

[87] Doi T, Makizako H, Tsutsumimoto K, Transitional status and modifiable risk of frailty in Japanese older adults: a prospective cohort study. Geriatr Gerontol Int. 2018;18(11):1562-1566. doi:10.1111/ggi.13525 30225955

[88] Hanlon P, Nicholl BI, Jani BD, Lee D, McQueenie R, Mair FS Frailty and pre-frailty in middle-aged and older adults and its association with multimorbidity and mortality: a prospective analysis of 493 737 UK Biobank participants. Lancet Public Health. 2018;3(7):e323-e332. doi:10.1016/S2468-2667(18)30091-4 29908859 PMC6028743

[89] Vetrano DL, Palmer K, Marengoni A, ; Joint Action ADVANTAGE WP4 Group Frailty and multimorbidity: a systematic review and meta-analysis. J Gerontol A Biol Sci Med Sci. 2018. 29726918 10.1093/gerona/gly110

[90] Ofori-Asenso R, Chin KL, Curtis AJ, Zomer E, Zoungas S, Liew D Recent patterns of multimorbidity among older adults in high-income countries. Popul Health Manag. 2019;22(2):127-137. doi:10.1089/pop.2018.0069 30096023

[91] Peralta M, Ramos M, Lipert A, Martins J, Marques A Prevalence and trends of overweight and obesity in older adults from 10 European countries from 2005 to 2013. Scand J Public Health. 2018;46(5):522-529. doi:10.1177/1403494818764810 29569525

[92] Samper-Ternent R, Al Snih S Obesity in older adults: epidemiology and implications for disability and disease. Rev Clin Gerontol. 2012;22(1):10-34. doi:10.1017/S0959259811000190 22345902 PMC3278274

[93] García-Esquinas E, José García-García F, León-Muñoz LM, Obesity, fat distribution, and risk of frailty in two population-based cohorts of older adults in Spain. Obesity (Silver Spring). 2015;23(4):847-855. doi:10.1002/oby.21013 25683024

[94] Stenholm S, Strandberg TE, Pitkälä K, Sainio P, Heliövaara M, Koskinen S Midlife obesity and risk of frailty in old age during a 22-year follow-up in men and women: the Mini-Finland follow-up survey. J Gerontol A Biol Sci Med Sci. 2014;69(1):73-78. doi:10.1093/gerona/glt052 23640762

[95] Feng Z, Lugtenberg M, Franse C, Risk factors and protective factors associated with incident or increase of frailty among community-dwelling older adults: a systematic review of longitudinal studies. PLoS One. 2017;12(6):e0178383. doi:10.1371/journal.pone.0178383 28617837 PMC5472269

[96] Ng TP, Feng L, Nyunt MS, Larbi A, Yap KB Frailty in older persons: multisystem risk factors and the Frailty Risk Index (FRI). J Am Med Dir Assoc. 2014;15(9):635-642. doi:10.1016/j.jamda.2014.03.008 24746590

[97] Espinoza SE, Fried LP Risk factors for frailty in the older adult. Clin Geriatr. 2007;15(6):37-44.

[98] Kojima G, Iliffe S, Walters K Smoking as a predictor of frailty: a systematic review. BMC Geriatr. 2015;15:131. doi:10.1186/s12877-015-0134-9 26489757 PMC4618730

[99] Siriwardhana DD, Hardoon S, Rait G, Weerasinghe MC, Walters KR Prevalence of frailty and prefrailty among community-dwelling older adults in low-income and middle-income countries: a systematic review and meta-analysis. BMJ Open. 2018;8(3):e018195. doi:10.1136/bmjopen-2017-018195 29496895 PMC5855322

[100] Franse CB, van Grieken A, Qin L, Melis RJF, Rietjens JAC, Raat H Socioeconomic inequalities in frailty and frailty components among community-dwelling older citizens. PLoS One. 2017;12(11):e0187946. doi:10.1371/journal.pone.0187946 29121677 PMC5679620

[101] Epping-Jordan JE, Pruitt SD, Bengoa R, Wagner EH Improving the quality of health care for chronic conditions. Qual Saf Health Care. 2004;13(4):299-305. doi:10.1136/qshc.2004.010744 15289634 PMC1743863

[102] Gordon EH, Peel NM, Samanta M, Theou O, Howlett SE, Hubbard RE Sex differences in frailty: a systematic review and meta-analysis. Exp Gerontol. 2017;89:30-40. doi:10.1016/j.exger.2016.12.021 28043934

[103] Puts MTE, Toubasi S, Andrew MK, Interventions to prevent or reduce the level of frailty in community-dwelling older adults: a scoping review of the literature and international policies. Age Ageing. 2017;46(3):383-392. 28064173 10.1093/ageing/afw247 PMC5405756

[104] Apóstolo J, Cooke R, Bobrowicz-Campos E, Effectiveness of interventions to prevent pre-frailty and frailty progression in older adults: a systematic review. JBI Database System Rev Implement Rep. 2018;16(1):140-232. doi:10.11124/JBISRIR-2017-003382 29324562 PMC5771690

[105] Chin A Paw MJ, van Uffelen JG, Riphagen I, van Mechelen W The functional effects of physical exercise training in frail older people: a systematic review. Sports Med. 2008;38(9):781-793. doi:10.2165/00007256-200838090-00006 18712944

[106] de Labra C, Guimaraes-Pinheiro C, Maseda A, Lorenzo T, Millán-Calenti JC Effects of physical exercise interventions in frail older adults: a systematic review of randomized controlled trials. BMC Geriatr. 2015;15:154. doi:10.1186/s12877-015-0155-4 26626157 PMC4667405

[107] Giné-Garriga M, Roqué-Fíguls M, Coll-Planas L, Sitjà-Rabert M, Salvà A Physical exercise interventions for improving performance-based measures of physical function in community-dwelling, frail older adults: a systematic review and meta-analysis. Arch Phys Med Rehabil. 2014;95(4):753-769.e3. doi:10.1016/j.apmr.2013.11.007 24291597

[108] Travers J, Romero-Ortuno R, Bailey J, Cooney MT Delaying and reversing frailty: a systematic review of primary care interventions. Br J Gen Pract. 2019;69(678):e61-e69. doi:10.3399/bjgp18X700241 30510094 PMC6301364

[109] Noubiap JJ, Balti EV, Bigna JJ, Echouffo-Tcheugui JB, Kengne AP Dyslipidaemia in Africa: comment on a recent systematic review: authors’ reply. Lancet Glob Health. 2019;7(3):e308-e309. doi:10.1016/S2214-109X(18)30517-5 30553650

