# Supplementary material for: Global Incidence of Frailty and Prefrailty Among Community-Dwelling Older Adults: A Systematic Review and Meta-analysis
Source: JAMA Netw Open. 2019 Aug 2;2(8):e198398. doi: 10.1001/jamanetworkopen.2019.8398 (PMC6681553; doi:10.1001/jamanetworkopen.2019.8398)
Supplement: Supplement. — eTable 1. Search Sequence for Ovid Medline Which Was Subsequently Adapted for Other Databases eFigure 1. A Map Showing the Geographical Spread of the Countries From Which Data Were Collected eFigure 2. Funnel Plot of Incidence Rates of Frailty Among Community-Dwelling Older Adults eFigure 3. Forest Plot of Incidence Rates of Frailty Among Community-Dwelling Older Adults When Factoring in Progression to Deaths in Studies With Death Data eFigure 4. Forest Plot of Incidence Rates of Frailty Among Community-Dwelling Older Adults When Limiting to Survivors in Studies With Death Data eFigure 5. Funnel Plot of Incidence Rates of Pre-frailty Among Community-Dwelling Older Adults eFigure 6. Forest Plot of Incidence Rates of Pre-frailty Among Community-Dwelling Older Adults When Factoring in Progression to Deaths in Studies With Death Data eFigure 7. Forest Plot of Incidence Rates of Pre-frailty Among Community-Dwelling Older Adults When Limiting to Survivors in Studies With Death Data eTable 2. Estimates From Leave-One-Out Sensitivity Analyses for the Incidence of Frailty eTable 3. Estimates of Leave-One-Out Sensitivity Analyses for the Incidence of Pre-frailty eReferences. [file jamanetwopen-2-e198398-s001.pdf]

# Supplementary Online Content

Ofori-Asenso R, Chin KL, Mazidi M, et al. Global incidence of frailty and prefrailty among community-dwelling older adults: a systematic review and meta-analysis. *JAMA Netw Open*. 2019;2(8):e198398. doi:10.1001/jamanetworkopen.2019.8398

**eTable 1.** Search Sequence for Ovid Medline Which Was Subsequently Adapted for Other Databases

**eFigure 1.** A Map Showing the Geographical Spread of the Countries From Which Data Were Collected

**eFigure 2.** Funnel Plot of Incidence Rates of Frailty Among Community-Dwelling Older Adults

**eFigure 3.** Forest Plot of Incidence Rates of Frailty Among Community-Dwelling Older Adults When Factoring in Progression to Deaths in Studies With Death Data

**eFigure 4.** Forest Plot of Incidence Rates of Frailty Among Community-Dwelling Older Adults When Limiting to Survivors in Studies With Death Data

**eFigure 5.** Funnel Plot of Incidence Rates of Pre-frailty Among Community-Dwelling Older Adults

**eFigure 6.** Forest Plot of Incidence Rates of Pre-frailty Among Community-Dwelling Older Adults When Factoring in Progression to Deaths in Studies With Death Data

**eFigure 7.** Forest Plot of Incidence Rates of Pre-frailty Among Community-Dwelling Older Adults When Limiting to Survivors in Studies With Death Data

**eTable 2.** Estimates From Leave-One-Out Sensitivity Analyses for the Incidence of Frailty

**eTable 3.** Estimates of Leave-One-Out Sensitivity Analyses for the Incidence of Pre-frailty

**eReferences.**

This supplementary material has been provided by the authors to give readers additional information about their work.

© 2019 Ofori-Asenso R et al. *JAMA Network Open*.

**eTable 1.** Search Sequence for Ovid Medline Which Was Subsequently Adapted for Other Databases

| | |
|---|---|
| 1 | exp Frailty |
| 2 | frailty.mp |
| 3 | exp Frail Elderly |
| 4 | Frail Elderly.sh,kf. |
| 5 | Frail Older People.mp. |
| 6 | Frailty syndrome.mp. |
| 7 | (Frailty *adj2* syndrome).mp. |
| 8 | (Frail* *or* geriatric syndrome* or geriatric disorder*).ti,ab. |
| 9 | ((elder* *or* old* *or* senior* *or* geriatric*) *adj4* function* *adj4* (declin* or impair*)).af. |
| 10 | 1 *or* 2 *or* 3 or 4 *or* 5 *or* 6 or 7 *or* 8 *or* 9 |
| 11 | Incidence.mp. or exp Incidence |
| 12 | Incidence*.mp. |
| 13 | Incidence.ti. |
| 14 | Incidence.sh. |
| 15 | (Epidemiology* or pattern* or cluster* or number*).ti. |
| 16 | Transition*.mp. |
| 17 | Trajectories.mp. |
| 18 | trajectory.mp. |
| 19 | 11 *or* 12 *or* 13 *or* 14 *or* 15 *or* 16 *or* 17 |
| 20 | 10 and 19 |

© 2019 Ofori-Asenso R et al. *JAMA Network Open*.

**eFigure 1**. A Map Showing the Geographical Spread of the Countries From Which Data Were Collected*

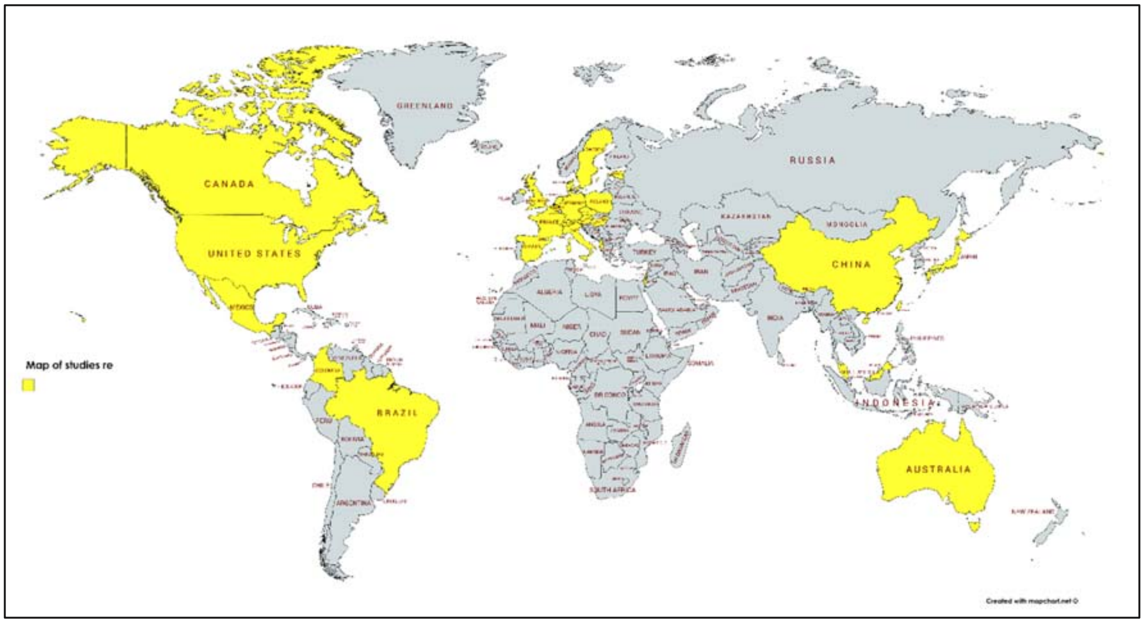


*Countries with included data are highlighted in yellow; map was created with *MapChart*.

**eFigure 2.** Funnel Plot of Incidence Rates of Frailty Among Community-Dwelling Older Adults

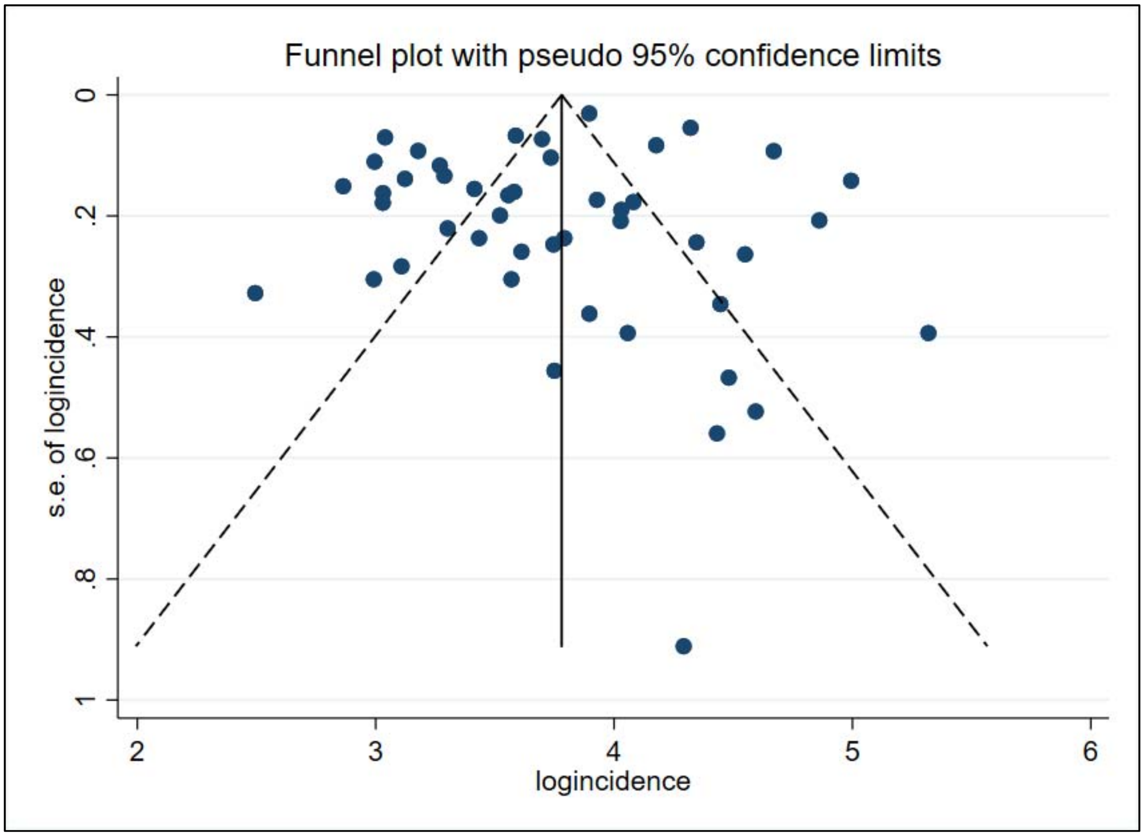


© 2019 Ofori-Asenso R et al. *JAMA Network Open*.

**eFigure 3.** Forest Plot of Incidence Rates of Frailty Among Community-Dwelling Older Adults When Factoring in Progression to Deaths in Studies With Death Data

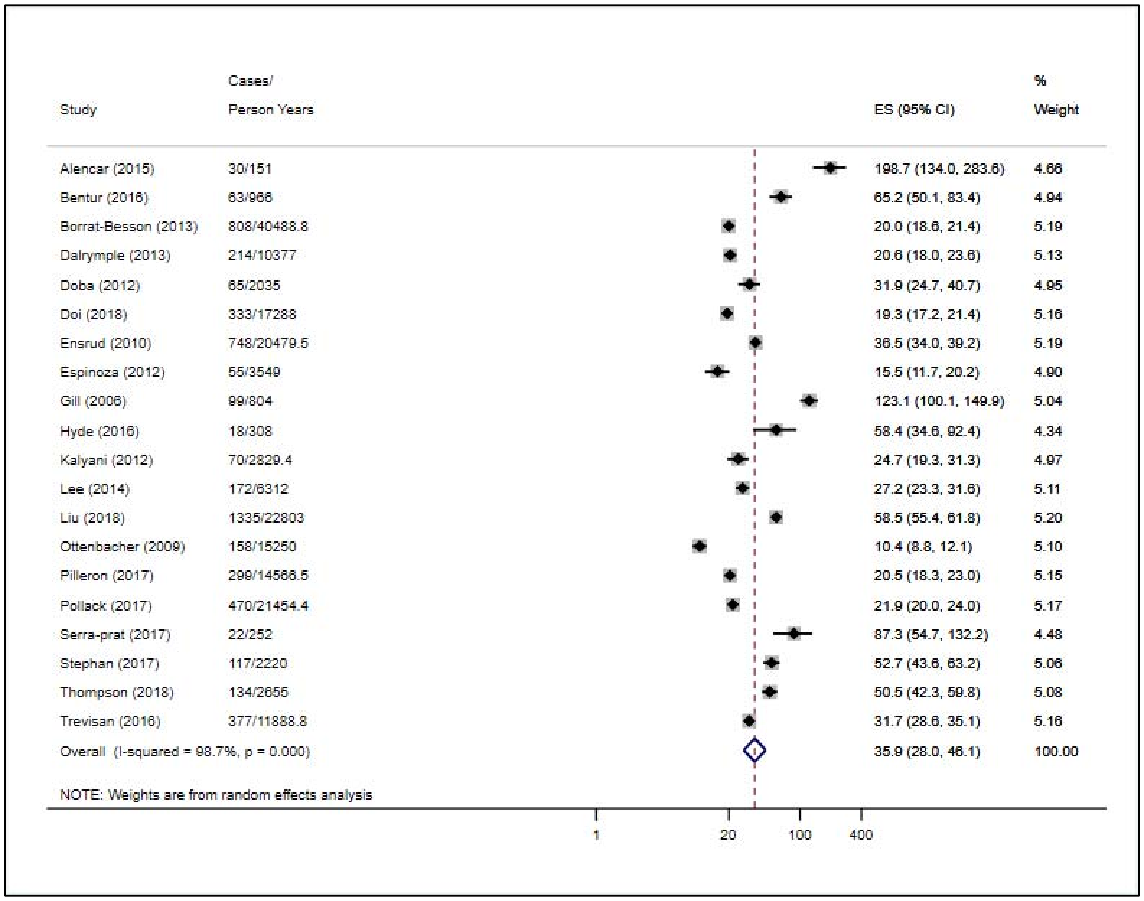


© 2019 Ofori-Asenso R et al. *JAMA Network Open*.

**eFigure 4.** Forest Plot of Incidence Rates of Frailty Among Community-Dwelling Older Adults When Limiting to Survivors in Studies With Death Data

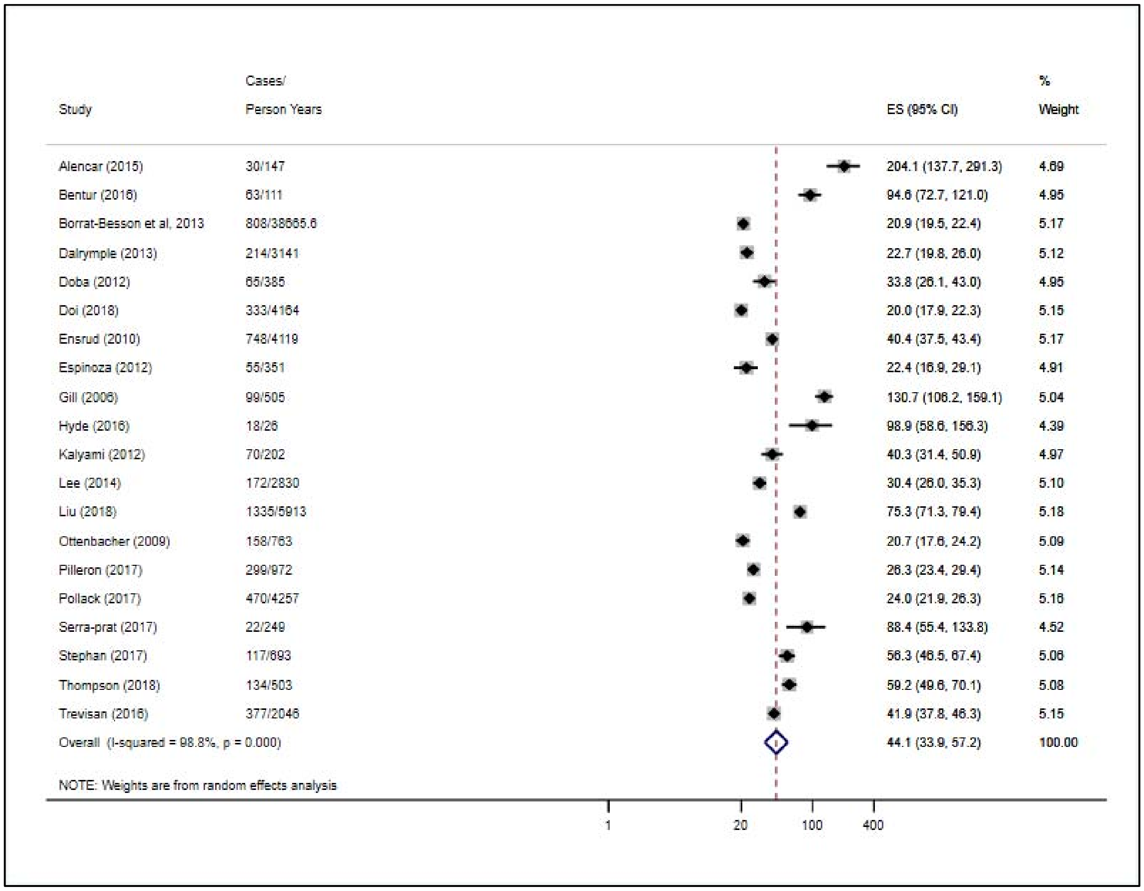


© 2019 Ofori-Asenso R et al. *JAMA Network Open*.

**eFigure 5.** Funnel Plot of Incidence Rates of Pre-frailty Among Community-Dwelling Older Adults

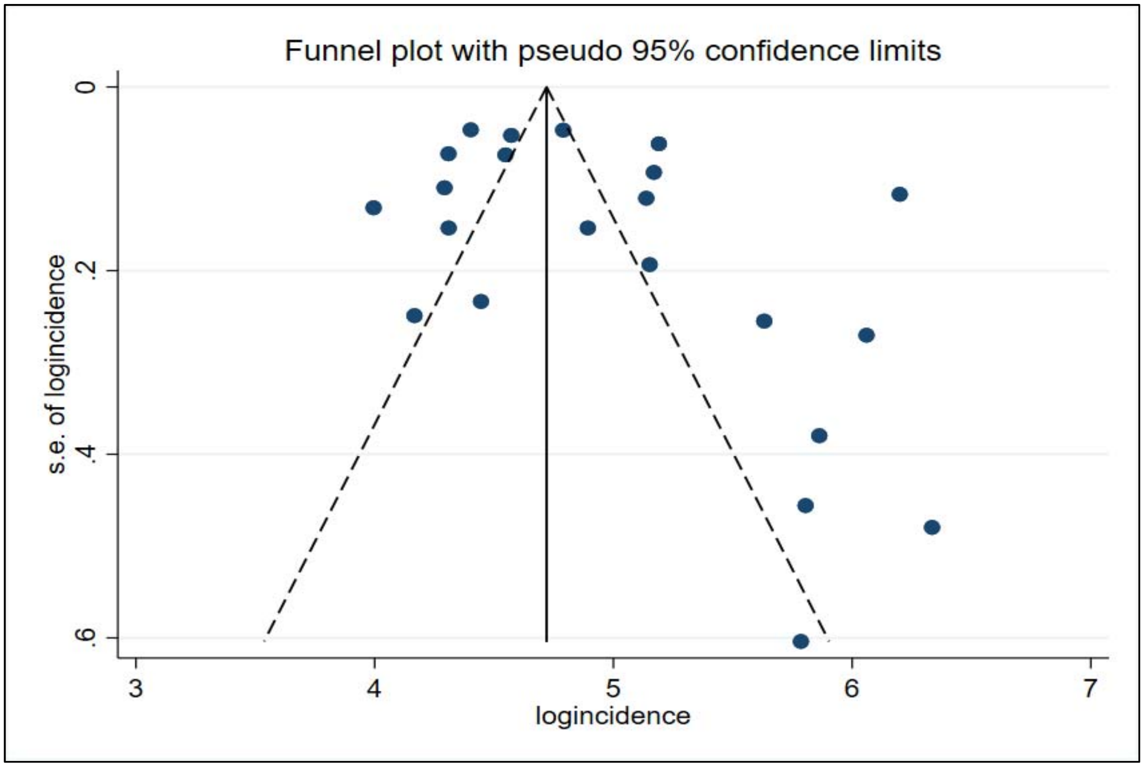


© 2019 Ofori-Asenso R et al. *JAMA Network Open*.

**eFigure 6.** Forest Plot of Incidence Rates of Pre-frailty Among Community-Dwelling Older Adults When Factoring in Progression to Deaths in Studies With Death Data

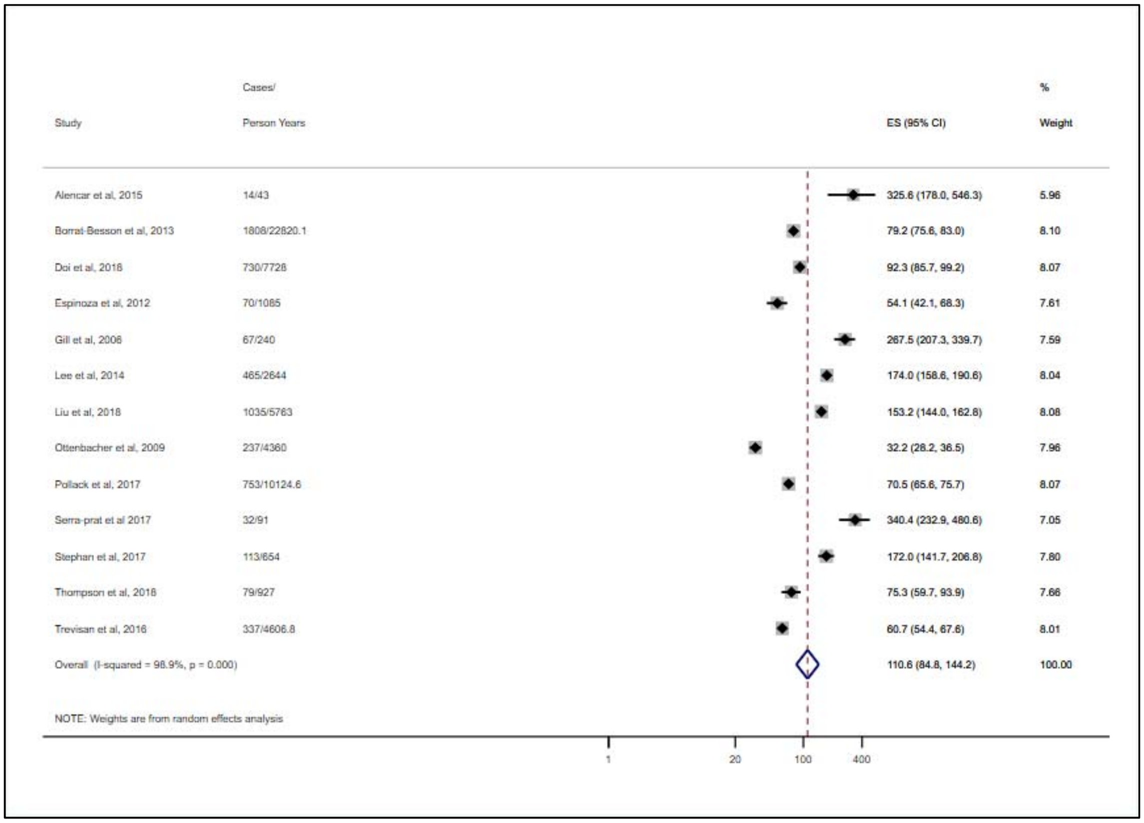


© 2019 Ofori-Asenso R et al. *JAMA Network Open*.

**eFigure 7.** Forest Plot of Incidence Rates of Pre-frailty Among Community-Dwelling Older Adults When Limiting to Survivors in Studies With Death Data

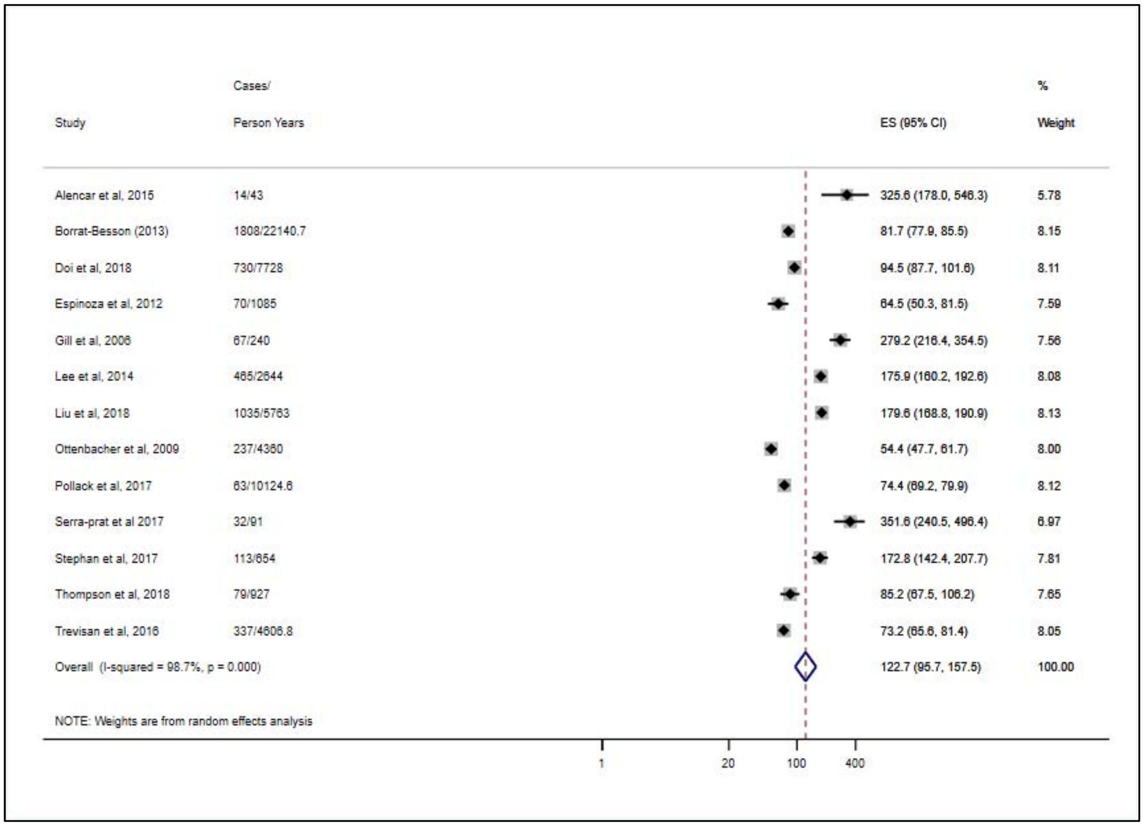


© 2019 Ofori-Asenso R et al. *JAMA Network Open*.

**eTable 2.** Estimates From Leave-One-Out Sensitivity Analyses for the Incidence of Frailty

| Study Omitted | Point estimate | Lower CI | Upper CI |
|---|---|---|---|
| Ahmad (2018)[1] | 43.4 | 37.2 | 50.6 |
| Alencar (2015)[2] | 42.0 | 36.1 | 48.8 |
| Ayers (2017a)[3] | 43.1 | 37.0 | 50.2 |
| Ayers (2017b)[3] | 42.7 | 36.7 | 49.8 |
| Baulderstone (2012)[4] | 44.3 | 38.1 | 51.4 |
| Bentur (2016)[5] | 42.6 | 36.6 | 49.6 |
| Borrat-Besson (2013)[6] | 44.1 | 38.1 | 51.0 |
| Castrejón-Pérez (2017)[7] | 43.2 | 37.1 | 50.4 |
| Chhetri (2018)[8] | 42.4 | 36.6 | 49.1 |
| Dalrymple (2013)[9] | 44.0 | 37.8 | 51.2 |
| Doba (2012)[10] | 43.5 | 37.3 | 50.7 |
| Doi (2018)[11] | 44.1 | 38.0 | 51.3 |
| Ensrud (2010)[12] | 43.5 | 37.1 | 50.9 |
| Espinoza (2012)[13] | 44.0 | 37.8 | 51.2 |
| Gale (2013)[14] | 43.8 | 37.6 | 51.1 |
| Garcia-Esquinas (2015)[15] | 43.6 | 37.4 | 50.8 |
| Garcia-Esquinas (2016)[16] | 42.8 | 36.8 | 49.8 |
| Gill (2006)[17] | 42.3 | 36.4 | 49.1 |
| Gnjidic (2012)[18] | 43.7 | 37.5 | 50.9 |
| Gomes (2018)[19] | 43.8 | 37.6 | 51.0 |
| Gruenewald (2013)[20] | 44.1 | 37.9 | 51.3 |
| Hyde (2016)[21] | 42.7 | 36.7 | 49.7 |
| Iwasaki (2018)[22] | 43.5 | 37.4 | 50.7 |
| Kalyami (2012)[23] | 43.3 | 37.2 | 50.5 |
| Kim (2017)[24] | 43.2 | 37.1 | 50.4 |
| Lanziotti (2015)[25] | 42.8 | 36.8 | 49.9 |
| Lee (2014)[26] | 43.7 | 37.5 | 51.0 |
| Liu (2018)[27] | 42.8 | 36.8 | 49.7 |
| Lorenzo-López (2019)[28] | 43.1 | 37.0 | 50.2 |
| Ottenbacher (2009)[29] | 44.1 | 37.9 | 51.3 |
| Pilleron (2017)[30] | 43.9 | 37.7 | 51.1 |
| Pollack (2017)[31] | 44.0 | 37.8 | 51.1 |
| Potier (2018)[32] | 43.0 | 37.0 | 50.1 |
| Ramsay (2018)[33] | 43.6 | 37.4 | 50.8 |
| Sandoval-Insausti (2016)[34] | 44.1 | 37.9 | 51.3 |
| Saum (2017)[35] | 43.6 | 37.4 | 50.8 |
| Semba (2006)[36] | 42.1 | 36.4 | 48.7 |
| Serra-prat (2017)[37] | 42.8 | 36.7 | 49.8 |
| Sha (2018)[38] | 43.6 | 37.2 | 51.0 |
| Stephan (2017)[39] | 43.1 | 37.0 | 50.2 |
| Swiecicka (2018)[40] | 44.6 | 38.3 | 51.8 |
| Thompson (2018)[41] | 43.1 | 36.9 | 50.2 |
| Trevisan (2016)[43] | 43.4 | 37.2 | 50.7 |
| Wang (2019)[44] | 43.4 | 37.2 | 50.5 |
| Woods (2005)[45] | 43.4 | 36.5 | 51.6 |
| Zaslavsky (2016)[46] | 43.0 | 36.8 | 50.1 |
| Combined | **43.4** | **37.3** | **50.4** |

© 2019 Ofori-Asenso R et al. *JAMA Network Open*.

**eTable 3.** Estimates of Leave-One-Out Sensitivity Analyses for the Incidence of Pre-frailty

| Study Omitted | Point estimate | Lower CI | Upper CI |
|---|---|---|---|
| Ahmad (2018)[1] | 140.1 | 118.0 | 166.2 |
| Alencar (2015)[2] | 146.5 | 119.5 | 179.5 |
| Borrat-Besson (2013)[6] | 155.7 | 125.7 | 192.8 |
| Doi (2018)[11] | 154.5 | 124.9 | 191.3 |
| Espinoza (2012)[13] | 156.8 | 127.7 | 192.4 |
| Gill (2006)[17] | 146.2 | 119.3 | 179.2 |
| Gomes (2018)[19] | 149.8 | 121.8 | 184.1 |
| Gruenewald (2013)[20] | 151.6 | 123.2 | 186.6 |
| Lanziotti (2015)[25] | 145.8 | 118.9 | 178.7 |
| Lee (2014)[26] | 149.5 | 121.7 | 183.7 |
| Liu (2018)[27] | 149.2 | 121.7 | 183.0 |
| Lorenzo-López (2019)[28] | 143.3 | 117.2 | 175.1 |
| Ottenbacher (2009)[29] | 158.3 | 129.4 | 193.7 |
| Pollack (2017)[31] | 156.2 | 126.9 | 192.1 |
| Potier (2018)[32] | 142.8 | 116.6 | 174.8 |
| Serra-prat (2017)[37] | 145.1 | 118.4 | 177.8 |
| Stephan (2017)[39] | 149.7 | 121.8 | 183.8 |
| Swiecicka (2018)[40] | 156.0 | 126.9 | 191.7 |
| Thompson (2018)[41] | 154.8 | 126.0 | 190.2 |
| Tom (2017a)[42] | 153.2 | 122.2 | 192.1 |
| Tom (2017b)[42] | 154.7 | 124.0 | 192.9 |
| Trevisan (2016)[43] | 156.2 | 127.1 | 192.0 |
| Combined | **150.6** | **123.3** | **184.1** |

© 2019 Ofori-Asenso R et al. *JAMA Network Open*.

## eReferences


1. Ahmad NS, Hairi NN, Said MA, et al. Prevalence, transitions and factors predicting transition between frailty states among rural community-dwelling older adults in Malaysia. *PloS one.* 2018;13(11):e0206445.

2. Alencar MA, Dias JMD, Figueiredo LC, Dias RC. Transitions in Frailty Status in Community-Dwelling Older Adults. *Topics in Geriatric Rehabilitation.* 2015;31(2):105-112.

3. Ayers E, Shapiro M, Holtzer R, Barzilai N, Milman S, Verghese J. Symptoms of Apathy Independently Predict Incident Frailty and Disability in Community-Dwelling Older Adults. *J Clin Psychiatry.* 2017;78(5):e529-e536.

4. Baulderstone L, Yaxley A, Luszcz M, Miller M. Diet Liberalisation in Older Australians Decreases Frailty without Increasing the Risk of Developing Chronic Disease. *J Frailty Aging.* 2012;1(4):174-182.

5. Bentur N, Sternberg SA, Shuldiner J. Frailty Transitions in Community Dwelling Older People. *The Israel Medical Association journal : IMAJ.* 2016;18(8):449-453.

6. Borrat-Besson C, Ryser V, Wernli B. Transitions between frailty states–a European comparison. 2013:175. In: Börsch-Supan A, Brandt M, Litwin H, Weber G, eds. *Active ageing and solidarity between generations in Europe: First results from SHARE after the economic crisis*. Germany: Hubert & Co. GmBH & Co.KG, Gottingen; 2013:175-185.

7. Castrejon-Perez RC, Jimenez-Corona A, Bernabe E, et al. Oral Disease and 3-Year Incidence of Frailty in Mexican Older Adults. *The journals of gerontology. Series A, Biological sciences and medical sciences.* 2017;72(7):951-957.

8. Chhetri JK, Zheng Z, Xu X, Ma C, Chan P. The prevalence and incidence of frailty in Pre-diabetic and diabetic community-dwelling older population: results from Beijing longitudinal study of aging II (BLSA-II). *BMC geriatrics.* 2017;17(1):47.

9. Dalrymple LS, Katz R, Rifkin DE, et al. Kidney function and prevalent and incident frailty. *Clin J Am Soc Nephrol.* 2013;8(12):2091-2099.

10. Doi T, Makizako H, Tsutsumimoto K, et al. Transitional status and modifiable risk of frailty in Japanese older adults: A prospective cohort study. *Geriatrics & gerontology international.* 2018;18(11):1562-1566.

11. Doba N, Tokuda Y, Goldstein NE, Kushiro T, Hinohara S. A pilot trial to predict frailty syndrome: the Japanese Health Research Volunteer Study. *Exp Gerontol.* 2012;47(8):638-643.

12. Ensrud KE, Ewing SK, Fredman L, et al. Circulating 25-Hydroxyvitamin D Levels and Frailty Status in Older Women. *Journal of Clinical Endocrinology & Metabolism.* 2010;95(12):5266-5273.

13. Espinoza SE, Jung I, Hazuda H. Frailty transitions in the San Antonio Longitudinal Study of Aging. *Journal of the American Geriatrics Society.* 2012;60(4):652-660.

14. Gale CR, Baylis D, Cooper C, Sayer AA. Inflammatory markers and incident frailty in men and women: the English Longitudinal Study of Ageing. *Age (Dordrecht, Netherlands).* 2013;35(6):2493-2501.

15. Garcia-Esquinas E, Jose Garcia-Garcia F, Leon-Munoz LM, et al. Obesity, Fat Distribution, and Risk of Frailty in Two Population-Based Cohorts of Older Adults in Spain. *Obesity.* 2015;23(4):847-855.


© 2019 Ofori-Asenso R et al. *JAMA Network Open*.

16. Garcia-Esquinas E, Rahi B, Peres K, et al. Consumption of fruit and vegetables and risk of frailty: a dose-response analysis of 3 prospective cohorts of community-dwelling older adults. *American Journal of Clinical Nutrition.* 2016;104(1):132-142.

17. Gomes CDS, Guerra RO, Wu YY, et al. Social and Economic Predictors of Worse Frailty Status Occurrence Across Selected Countries in North and South America and Europe. *Innov Aging.* 2018;2(3):igy037.

18. Gill TM, Gahbauer EA, Allore HG, Han L. Transitions between frailty states among community-living older persons. *Archives of internal medicine.* 2006;166(4):418-423.

19. Gnjidic D, Hilmer SN, Blyth FM, et al. High-risk prescribing and incidence of frailty among older community-dwelling men. *Clinical pharmacology and therapeutics.* 2012;91(3):521-528.

20. Gruenewald TL, Seeman TE, Karlamangla AS, Sarkisian CA. Allostatic load and frailty in older adults. *J Am Geriatr Soc.* 2009;57(9):1525-1531.

21. Hyde Z, Flicker L, Smith K, et al. Prevalence and incidence of frailty in Aboriginal Australians, and associations with mortality and disability. *Maturitas.* 2016;87:89-94.

22. Iwasaki M, Yoshihara A, Sato M, et al. Dentition status and frailty in community-dwelling older adults: A 5-year prospective cohort study. *Geriatr Gerontol Int.* 2018;18(2):256-262.

23. Kalyani R, Tian J, Xue Q, et al. Hyperglycemia and incidence of frailty and lower extremity mobility limitations in older women. *Journal - American Geriatrics Society.* 2012;60(9):1701-1707.

24. Kim M, Suzuki T, Kojima N, et al. Association Between Serum beta2 -Microglobulin Levels and Prevalent and Incident Physical Frailty in Community-Dwelling Older Women. *Journal of the American Geriatrics Society.* 2017;65(4):e83-e88.

25. Lanziotti Azevedo da Silva S, Campos Cavalcanti Maciel A, de Sousa Maximo Pereira L, Domingues Dias JM, Guimaraes de Assis M, Correa Dias R. Transition Patterns of Frailty Syndrome in Comunity-Dwelling Elderly Individuals: A Longitudinal Study. *The Journal of frailty & aging.* 2015;4(2):50-55.

26. Lee JSW, Auyeung TW, Leung J, Kwok T, Woo J. Transitions in frailty states among community-living older adults and their associated factors. *Journal of the American Medical Directors Association.* 2014;15(4):281-286.

27. Liu ZY, Wei YZ, Wei LQ, et al. Frailty transitions and types of death in Chinese older adults: A population-based cohort study. *Clinical Interventions in Aging.* 2018;13:947-956.

28. Lorenzo-Lopez L, Lopez-Lopez R, Maseda A, Bujan A, Rodriguez-Villamil JL, Millan-Calenti JC. Changes in frailty status in a community-dwelling cohort of older adults: The VERISAUDE study. *Maturitas.* 2019;119:54-60.

29. Ottenbacher KJ, Graham JE, Al Snih S, et al. Mexican Americans and frailty: findings from the Hispanic established populations epidemiologic studies of the elderly. *American journal of public health.* 2009;99(4):673-679.

30. Pilleron S, Ajana S, Jutand MA, et al. Dietary Patterns and 12-Year Risk of Frailty: Results From the Three-City Bordeaux Study. *Journal of the American Medical Directors Association.* 2017;18(2):169-175.

31. Pollack LR, Litwack-Harrison S, Cawthon PM, et al. Patterns and Predictors of Frailty Transitions in Older Men: The Osteoporotic Fractures in Men Study. *Journal of the American Geriatrics Society.* 2017;65(11):2473-2479.

© 2019 Ofori-Asenso R et al. *JAMA Network Open*.

32. Potier F, Degryse JM, Bihin B, et al. Health and frailty among older spousal caregivers: an observational cohort study in Belgium. *BMC geriatrics.* 2018;18(1):291.

33. Ramsay SE, Papachristou E, Watt RG, et al. Influence of Poor Oral Health on Physical Frailty: A Population-Based Cohort Study of Older British Men. *Journal of the American Geriatrics Society.* 2018;66(3):473-479.

34. Sandoval-Insausti H, Perez-Tasigchana RF, Lopez-Garcia E, Garcia-Esquinas E, Rodriguez-Artalejo F, Guallar-Castillon P. Macronutrients Intake and Incident Frailty in Older Adults: A Prospective Cohort Study. *The journals of gerontology. Series A, Biological sciences and medical sciences.* 2016;71(10):1329-1334.

35. Saum K-U, Schoettker B, Meid AD, et al. Is Polypharmacy Associated with Frailty in Older People? Results From the ESTHER Cohort Study. *Journal of the American Geriatrics Society.* 2017;65(2):E27-E32.

36. Swiecicka A, Eendebak R, Lunt M, et al. Reproductive Hormone Levels Predict Changes in Frailty Status in Community-Dwelling Older Men: European Male Ageing Study Prospective Data. *The Journal of clinical endocrinology and metabolism.* 2018;103(2):701-709.

37. Semba RD, Bartali B, Zhou J, et al. Low serum micronutrient concentrations predict frailty among older women living in the community. *Journals of Gerontology Series A: Biological Sciences & Medical Sciences.* 2006;61(6):594-599.

38. Serra-Prat M, Papiol M, Vico J, Palomera E, Arús M, Cabré M. Incidence and Risk Factors for Frailty in the Community-Dwelling Elderly Population. A Two-Year Follow-Up Cohort Study. *Journal of Gerontology and Geriatric Research.* 2017;6(452).

39. Shah M, Paulson D, Vu N. Alcohol Use and Frailty Risk among Older Adults over 12 Years: The Health and Retirement Study. *Clinical Gerontologist.* 2018;41(4):315-325.

40. Stephan AJ, Strobl R, Holle R, et al. Male sex and poverty predict abrupt health decline: Deficit accumulation patterns and trajectories in the KORA-Age cohort study. *Prev Med.* 2017;102:31-38.

41. Thompson MQ, Theou O, Adams RJ, Tucker GR, Visvanathan R. Frailty state transitions and associated factors in South Australian older adults. *Geriatr Gerontol Int.* 2018;18(11):1549-1555.

42. Tom S, Wyman A, Woods N, et al. Regional Differences in Incident Prefrailty and Frailty. *Journal of Women's Health.* 2017;26(9):992-998.

43. Trevisan C, Veronese N, Maggi S, et al. Marital Status and Frailty in Older People: Gender Differences in the Progetto Veneto Anziani Longitudinal Study. *Journal of women's health (2002).* 2016;25(6):630-637.

44. Woods NF, LaCroix AZ, Gray SL, et al. Frailty: emergence and consequences in women aged 65 and older in the Women's Health Initiative Observational Study. *J Am Geriatr Soc.* 2005;53(8):1321-1330.

45. Wang M, T L, Li c, et al. Frailty, transition in frailty status and allcause mortality in older adults of a Taichung community-based population. *BMC geriatrics.* 2019;19(26).

46. Zaslavsky O, Walker RL, Crane PK, Gray SL, Larson EB. Glucose Levels and Risk of Frailty. *The journals of gerontology. Series A, Biological sciences and medical sciences.* 2016;71(9):1223-1229

© 2019 Ofori-Asenso R et al. *JAMA Network Open*.
